# Multiscale off-fault brecciation records coseismic energy budget of principal fault zone

**DOI:** 10.1038/s41598-024-62838-x

**Published:** 2024-05-27

**Authors:** Geri Agroli, Atsushi Okamoto, Masaoki Uno, Noriyoshi Tsuchiya

**Affiliations:** 1 Graduated School of Environmental Studies, Tohoku University, Sendai, 980-8579 Japan; 2 National Institute of Technology, Hachinohe College, Hachinohe, Aomori 039-1192 Japan

**Keywords:** Structural geology, Petrology

## Abstract

Breccia and pulverized rock are typical textures in off-fault damage adjacent to a main seismogenic zone. Previously, by estimating the energy required to advance the rupture in this zone using particle size distribution at sub-millimeter/micrometer scales, we could constrain the energy budget during coseismic events. However, whether microscopic estimation is sufficient to capture surface energy fragmentation during an earthquake and the effect of measurement scale variation on calculation of co-seismic energy partitioning remained unclear. Here, we investigated the mechanism of coseismic off-fault damage based on field and microstructural observations of a well-exposed breccia body in Ichinokawa, Japan. We used in situ clast measurements coupled with thin-section analysis of breccia clasts to estimate the energy budget of the damage zone adjacent to the principal slip zone of the Median Tectonic Line (MTL). The total surface energy density and corresponding surface energy per unit fault for a width of ~ 500 m of the dynamical damage zone were estimated. The moment magnitude estimated based on surface energy was 5.8–8.3 Mw. In Ichinokawa, off-fault fragmentation is initiated by coseismic activity and is followed by fluid activity. Under dynamic fragmentation conditions, the scale is important to calculate the surface energy.

## Introduction

The damage zone in the vicinity of a principal fault is considered as a key geological feature that encompasses various processes like faulting-induced deformation, strain distribution, regional deformation history, earthquake rupture propagation, associated seismic risk, and crustal fluid permeability^1–5^. The pervasive fracture development in the off-fault region indicates that the energy was consumed during the slip event and potentially linked to coseismic activity^6^. The prevailing paradigm for the coseismic slip is the presence of pseudotachylite indicating the frictional melting with a high slip rate (1–0.1 m/s) during slip phenomena^7–9^. Yet, as the number of studies grows, the fast rupture is considered to be a proxy for earthquakes that occur at extreme stress conditions^7^. The signatures of fast rupture include rock pulverization^10^ and periodic injection of fluids^11–13^.

When an earthquake occurs, the released elastic energy is translated into ground shaking (*U*_*rad*_), frictional heat (*U*_*fh*_), and increase in the area of surface energy (*U*_*sa*_)^14–16^. The surface fracture energy is an important energy input that is responsible for the initiation and advancement of rupture in the off-fault region^17–19^. The previous study suggests that surface energy accounts for 6% of earthquake breakdown work. This surface energy is responsible for enhancing off-fault damage and contributes significantly to calculating the overall energy balance during co-seismic rupture^6,14,20^. Surrounding active and mature continental strike-slip faults, the surface energy is manifested as well-preserved pervasive fragmentation of rocks that occur within the off-fault region (tens to hundreds of meters in scale) as pulverized rocks^21–28^. Experiments and seismological modeling have demonstrated that pulverized rocks require high to moderate strain rates, which can be attained through either single or successive loading of intact rock^10,29–33^. This loading presumably mimics the pulse of the surface energy during the seismic event, but only a few studies estimate this energy budget directly from the exhumed fault. Here we use fragmented rocks at the field (in situ) and thin-section scales to reconstruct the dynamics of the energy budget experienced by the rock and bridge the gap between the field and experimental result^34,35^.

We performed a multidisciplinary study of pulverized pelitic schists exposed in the off-fault region of the Median Tectonic Line (MTL) in Ichinokawa, Japan. Based on the texture and structure of breccia combined with particle size distribution (PSD) at various observation scales, we redefine the dynamics of the brecciation mechanism and its relationship with the MTL^36^. We performed calculations of total surface energy based on the size of breccia fragments at the outcrop and thin-section scales. Our findings reveal a novel rupture mechanism in the off-fault region of the MTL^14,37^ and constrain the scaling effect on the estimation of the earthquake energy budget. Our analyses reveal unique fragmentation behavior (pulverization), earthquake energy estimation, fluid circulation, and mineralization in the off-fault region. Therefore, Ichinokawa can serve as a reference site for the study of such phenomena.

## Results and discussion

### Off fault pulverized rock associated with principal slip zone

**Figure 1:**
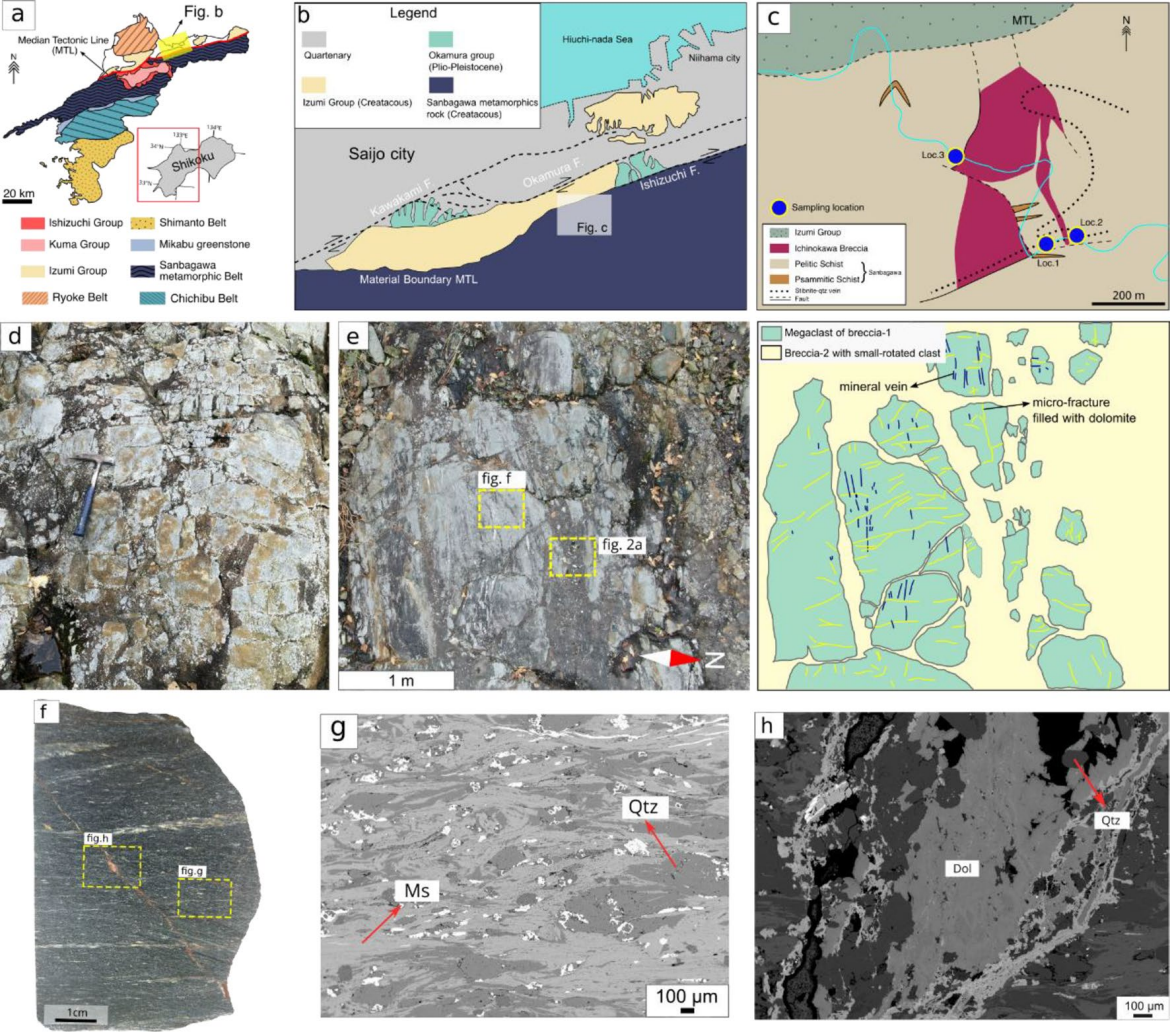
Representative field occurrences of breccia-1 in Ichinokawa. (**a**) Geological map of the Shikoku. (**b**) Geological maps and distribution of MTL segments around Saijo. (**c**) District geology of Ichinokawa areas where the apparent relationship between the MTL and Ichinokawa breccia in terms of distances are observed. (**d**) Crackle breccia with minimum clast-rotation; breccia-1 (bx-1). (**e**) Aerial photograph and schematic illustration showing the relationship between bx-1 and breccia-2 (bx-2), where the pervasive bx-2 intrudes the mega clast of bx-1 with the respective mineral vein (qtz) parallel to schistosity and microfracture filled with dolomite. (**f**) Hand specimen of low-grade Sanbagawa pelitic schist and (**g**) its typical mineral assemblages that become a clast component of Ichinokawa breccia. (**h**) Mode I microfracture in pelitic schist. The fracture filled by dolomite and a lower quantity of quartz observed under backscatter detector image, which indicates the earliest generation of the matrix after the formation of bx-1.

The Sanbagawa belt is a Cretaceous high-pressure metamorphosed accretionary complex consisting mainly of pelitic, psammitic, and mafic schists with minor quantities of metachert, expressing the subduction of marine sediment deposited upon the basaltic oceanic crust^38^. Across Shikoku Island, the Sanbagawa belt and the young accretionary complex in the north are separated by a 300 km-long arc-parallel tectonic fault of the Median Tectonic Line (MTL)^39^ (Fig. 1a). Right-lateral slip displacement, recorded in the latest history, contributes to the deformation of adjacent rock in terms of brittle failure including brecciation^40,41^. The MTL is divided into three segments and 12 subsegments^39,42^. The eastern segment is subdivided into the Kongo, Gojodani, and Negoro fault segments with an average slip rate of 1–3 mm/yr. The western part consists of the Iyo and Kawakami fault segments with an average slip rate of 0.5–5 mm/yr. The central segment, from east to west, is subdivided into the Naruto, Tsunden, Chichio, Mino, Ikeda, Ishizhuci, and Okamura fault segments with a slip rate of 5–9 mm/yr.

The Ichinokawa breccia body is located in central Shikoku approximately 3 km south of Saijo City within the off-fault region of the Okamura/Ishizuchi segment of MTL (Fig. 1b). The lenticularly restricted breccia body is perpendicular to the MTL with a dimension of 200 × 400 m consisting of low-grade Sanbagawa pelitic schist. It is surrounded by intact pelitic schist with lesser occurrences of psammitic schist. In the north, the rock body is juxtaposed with the Upper Cretaceous sediment formation of Izumi Group bounded by the ENE–WSW trending MTL (Fig. 1c and Supplementary Fig. 1a). The distance between the MTL and breccia is 50–650 m, representing the width of the damage zone (Supplementary Fig. 1b). The strike of the breccia and clast elongation are subparallel to the MTL (Supplementary Fig. 1c); however, in another outcrop, in situ measurement of the strike of the fault-filling breccia shows that it is perpendicular to the MTL (Supplementary Fig. 1d,e).

The pervasive brecciation composed of monomict and polymict breccia is well exposed in location 1 near the Senga-ko adit. Subsequently this breccia is cut by mineralized stibnite-quartz vein (Supplementary Fig. 2). The breccia-1 (bx-1) is clast supported breccia and is composed solely of pelitic schist clast with a size of up to 5 m (monomict; Fig. 1d, e). The pelitic schist has typical mineral assemblages of muscovite and quartz with EW sub-horizontal lineation of the Sanbagawa belt (Fig. 1f,g). The alteration is absent from the clast and the clast also shows little or no rotational block as well as dilatational shear deformation with well-preserved metamorphic lineation. Clasts are mostly angular and exhibit a crackle texture with non-systematically oriented micro- to macro fractures separating the clast. The fine microfracture cuts the lineation and mineral vein filled with dolomite with lesser quartz (Fig. 1h).

The Ichinokawa, which is located tens to hundreds of meters away from the MTL, is an ideal distance for causing coseismic fragmentation^29^. This process involves minimal clast rotation and resembles characteristics of rock pulverization. Aben et al.^29^, reported the in situ location of the observed pulverized outcrop with respect to the fault in ranges 15 cm–400 m for the case of crystalline rock. The various strike orientations of breccia (Supplementary Fig. 1e) and the complex deformation history of the MTL^40,41^, coupled with a significant seismic event of magnitude 7–8^43–45^ preceding the formation of Ichinokawa, support the idea of the MTL contributing to coseismic rupturing. Moreover, the theory of asymmetrical damage involving bi-material under subshear conditions applies here, where fracture/breccia is more pronounced in stiff rock (pelitic schist of Sanbagawa) compared to compliant sediment (Izumi formation)^23,46,47^. This asymmetrical damage in Ichinokawa creates a narrow and local breccia body that resembles the pulverized outcrop along the San Andreas Fault^34,35^, while the Izumi Formation accommodates the slip event through boudinage formation^48^. Finally, The unrotated block of pelitic schist and the absence of alteration around the clast also indicate that mechanical processes mainly govern the comminution which is most likely due to coseismic activity^49^. Judging from regional geology, kinematic analysis, and clast characteristics, we suggest that the formation of bx-1 is most likely linked to dynamic fragmentation related to the principal fault of MTL or as a rock pulverization.

### Fluidization and signature of decarbonation during seismic slip

**Figure 2:**
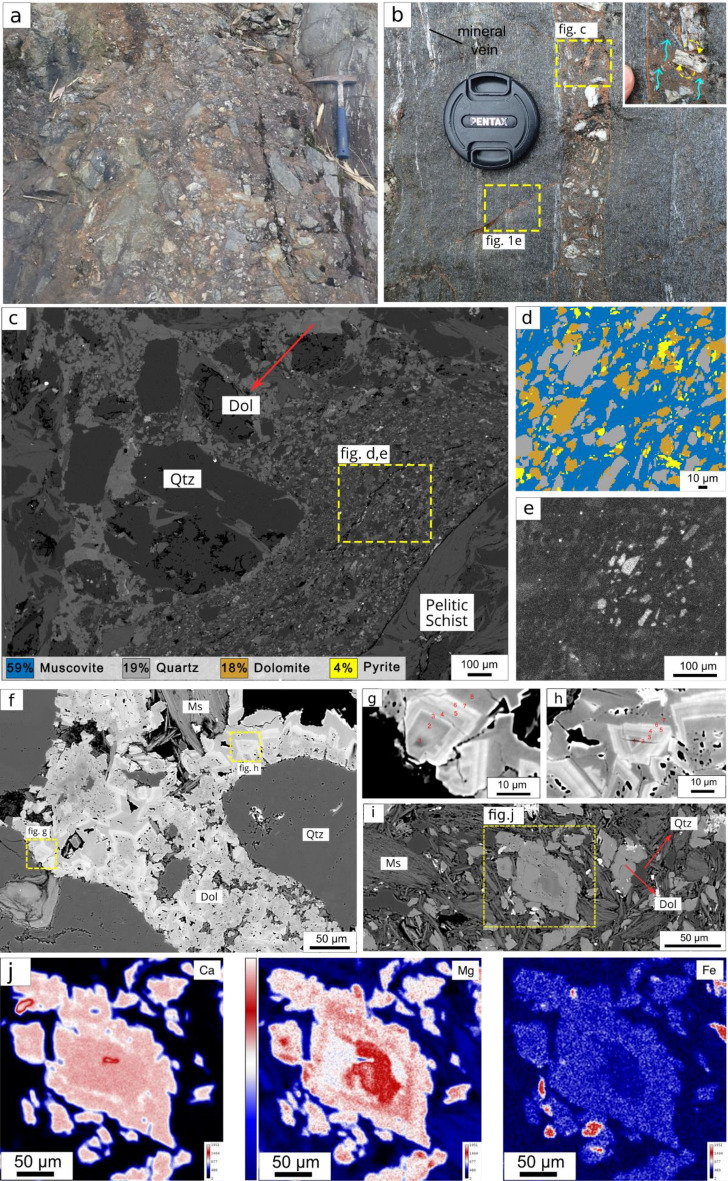
Textural and microscopic observations of breccia-2. (**a**) Breccia-2 with chaotic polymict clasts. (**b**) Various breccia widths and sharp boundaries are the unique features of bx-2. (**c**) The relationship between clast and matrix in bx-2. (**d**) Matrix–mineral phase maps and their relative abundances estimated using XmapTools^94^. (**e**) SEM-CL of the quartz matrix that depicts the crystallographic orientation of the quartz. (**f**) Dolomite coating of the quartz clast and (**g**, **h**) show the oscillatory zoning with different gray bands in the backscatter detector image. (**i**) Perfectly double-terminated dolomite crystal. (**j**) Elemental mapping of dolomite shows distinct characteristics between the core and rim.

The cohesive pulverized rock observed in Ichinokawa, unlike the typical incohesive pulverized rocks studied so far^25^, can be explained by the presence of breccia-2 (bx-2), which occupies the same outcrop as bx-1 and occurs as an injection-like breccia with various breccia pipe widths ranging from 5 cm to 1 m (Fig. 2a,b). The bx-2 is matrix-supported with polymict clast consisting of pelitic schist and a metamorphic mineral-bearing vein that is derived from bx-1 (Fig. 1e). The clast is sub-rounded to sub-angular with minimum corrosive wear observed from the clast (Fig. 2b,c). The average size of the clast is 20 mm at outcrop and 2.6 mm at thin-section scales, with a lower quantity of sub-millimeter particle size observed in backscatter detector (BSE) images (Fig. 2c). The clast is intensely rotated relative to the orientation of mineral vein and schistosity of pelitic schist and it shows a fluidized texture (Fig. 2b).

The matrix consists of muscovite, quartz, dolomite, and several sulfide minerals such as pyrite, arsenopyrite, and stibnite/sulfosalt, which make up 59, 19, 18, and 9%, respectively (Fig. 2d,e). Dolomite constitutes up to 40% (Supplementary Fig. 3a) of the matrix, with two observed precipitation modes: 1) the dolomite encloses another component similar to that of a cockade with distinct oscillatory zoning (Fig. 2f–h). It is distributed evenly along with another matrix component (Fig. 2i). The dolomite exhibits compositional zoning, with the core having higher magnesium and calcium but lower iron, whereas the rim has a higher iron content, giving rise to the reddish matrix color (Fig. 2j and Supplementary Fig. 3b,c). The quartz matrix shows a similar orientation of the c-axis under the BSE image (Fig. 2c) and under cathodoluminescence (CL) the quartz matrix has a high CL band compared to the low CL band of quartz clast (Fig. 2e).

The significant quantity of dolomite and carbonic fluid inclusions indicates the existence of a CO_2_-rich fluid and its circulation during co-seismic, even though the source of the fluid remains unknown (Supplementary Text). The presence of dolomite with a similar texture was observed in Hiyabashi borehole targeting the active Nojima fault which is related to the recent Kobe earthquake^50^. Where they suggest it is due to co-seismic hydrofracturing and subsequent fluid circulation, a rapid drop of fluid pressure accelerates the nucleation of the dolomite^51^. Moreover, The abundance of CO_2_-bearing fluid along the MTL is manifested as a calcite/dolomite vein within Sanbagawa^52,53^, dolomite-bearing stibnite deposits, and a modern CO_2_ hot spring, which indicates CO_2_ activity derived from hydrothermal sources^54,55^ (Supplementary Fig. 4). The low concentration CO_2_ fluid also observed within the quartz-bearing stibnite, as qtz-stibnite truncate the breccia^36^ indicates the remnant fluid also operate during the mineralization processes.

The difference of CL intensities between qtz matrix and clast also suggests the qtz clast is precipitate from silica-saturated fluid^56^ or some degree of reaction occurred between the rising fluid and pelitic schist (Fig. 2e). The zoning we observed from sulfide minerals also supports the notion that the fluidization was caused by pressure fluctuation and change in the fluid-flow regime during coseismic and interseismic events (Supplementary Fig. 5)^57,58^. Based on the clast characteristics and fluid involvement during co-seismic processes, we then inferred that fluidization processes dictate the formation of bx-2.

### Particle size distribution and dynamics of off-fault brecciation

PSD analysis is a powerful tool for studying fragmented rock or other materials^59,60^. The dimensionless D value or fractal dimension represents various mechanisms of clast fragmentation and size reduction (comminution) as mentioned by various authors^61–63^. Particularly in the brecciated system, the D value is a function of energy input applied to the rock during breccia formation^49^, and it has high and low values for high and low energy input processes, respectively. However, the fundamental process of both characterizations is that one emphasizes fault-related fragmentation, whereas the other indicates processes related to hydrothermal systems. Therefore, high and low D values depend on the process.

**Figure 3:**
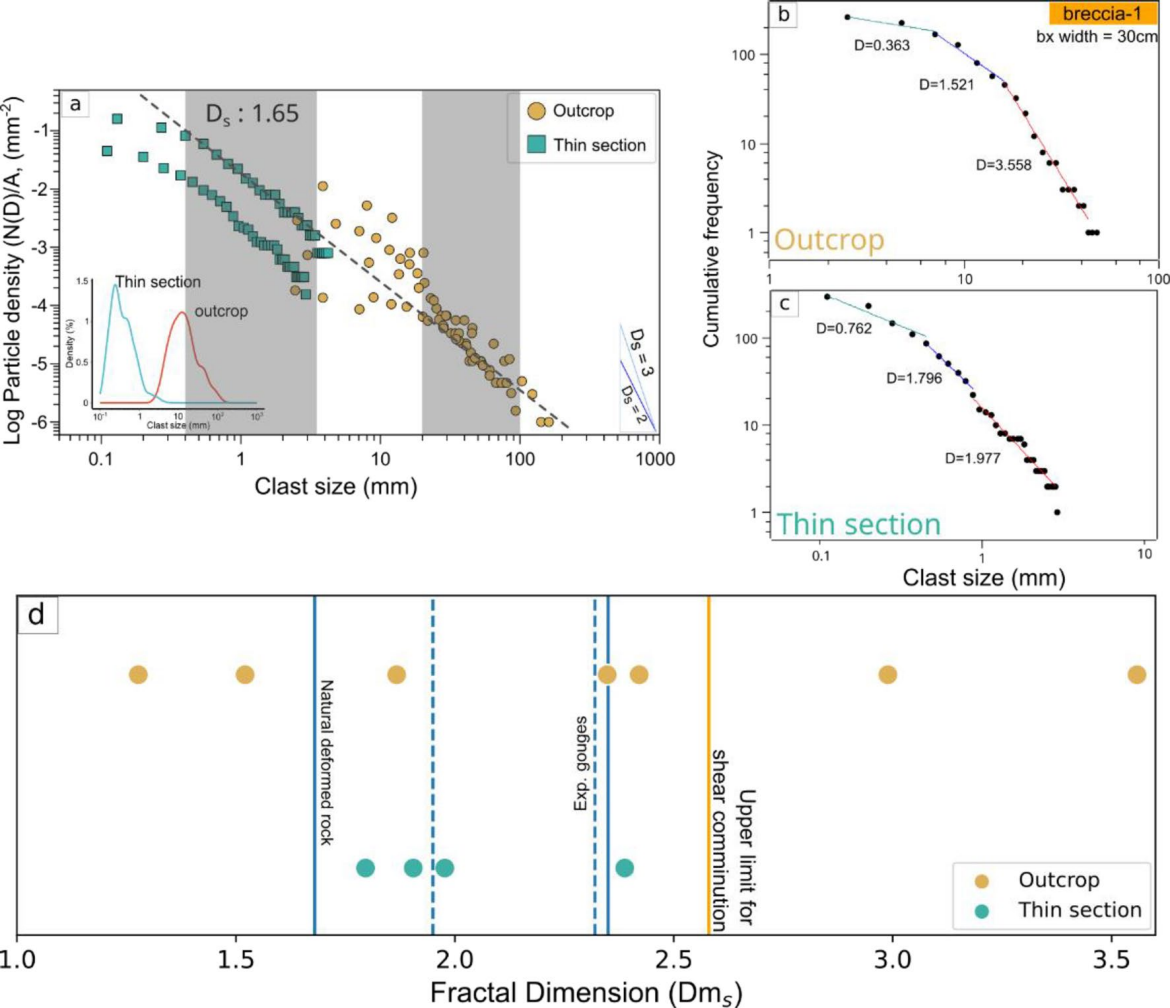
Particle size distribution (PSD). (**a**) Scale-integrated distribution of clast size as a function of clast density. The N(D) is the number of grains normalized to the measurement area at the outcrop and thin-section scales. Distributions of clasts at the (**b**) outcrop and (**c**) thin-section scales. (**d**) The relationship between fractal dimensions (D) and various rock deformation mechanisms from natural observations to experimental cases modified after Muto et al.^24^.

The integrated PSD in Ichinokawa (Fig. 3) follows a power law distribution with a slope of 1.65 for the clast spanning 0.3–3.5 mm for the thin section and 20–100 mm for the outcrop (indicated as the gray area). The clast size varies between 5 and 500 mm, and this wide range of clast sizes represents bx-1 and bx-2 at different observation scales. Under the thin section, the particle density has two major trends due to the difference in sampling locations, wherein the high particle density corresponds to bx-1 (Fig. 1c). The discrepancies in particle density at the outcrop scale is a function of the width of the breccia pipe. According to the density plot, the clast sizes at the outcrop and thin-section scales have mean clast diameters of 20.4 and 0.483 mm, respectively, with modal values of the distribution curves of 0.25 and 12.6 mm for the thin-section and outcrop, respectively (Fig. 3a).

In addition, the distribution of individual samples shows a relatively higher fractal dimension (D value) with more than one size distribution gradient. In the outcrop scale, bx-1 has the highest D value of 3.5, probably due to less variation in the clast size, as the rock was subjected to initial fragmentation (Fig. 3b and Supplementary Fig. 6a). The bx-2 has a fractal dimension of 1.2–2.9, with a positive correlation with the size of the breccia pipe. The distribution of clasts at the thin-section scale is relatively less heterogeneous, with the fractal dimension of 1.8–2.3 (Fig. 3c and Supplementary Fig. 6b).

The grain size from the most pulverized outcrops is smaller than 0.1 mm^11^. However, the situation is problematic in Ichinokawa, where most grain size is above 0.1 mm. Nevertheless, Agosta et al.^64^ have documented millimeter-sized grains in pulverized carbonate. Additionally, certain dynamic loading experiments have shown the evolution of pulverization^65^, achievable at strain rates lower than the previously proposed threshold (> 150 s−1)^31^. This lower strain rate can also lead to grain sizes on the micrometer scale. This is also supported by the D value in Ichinokawa which is in ranges 1.2–3.5 at both the outcrop and thin-section scales (Fig. 3d). Most of the D values coincide with the fractal dimension of natural and experimental fault gouges^24,66,67^. The distribution of fragments of bx-1 and bx-2 with the widest apertures correspond with high D values, surpassing the theoretical boundary of shear comminution processes^68^, which can be attained by extensive fragmentation and impact loading such as pervasive fracturing and rock pulverization^24,31,62,63^. The aperture of the conduit also contributes to the high-energy input during the dynamics of brecciation^49,69^. A fractal dimension of < 1.68 implies that fragmentation proceeds to the next step with a lower energy input associated with propagation, mechanical/chemical wearing, dilatation, and fragmentation caused by fluids, as observed in Ichinokawa^36,49,61^.

**Figure 4:**
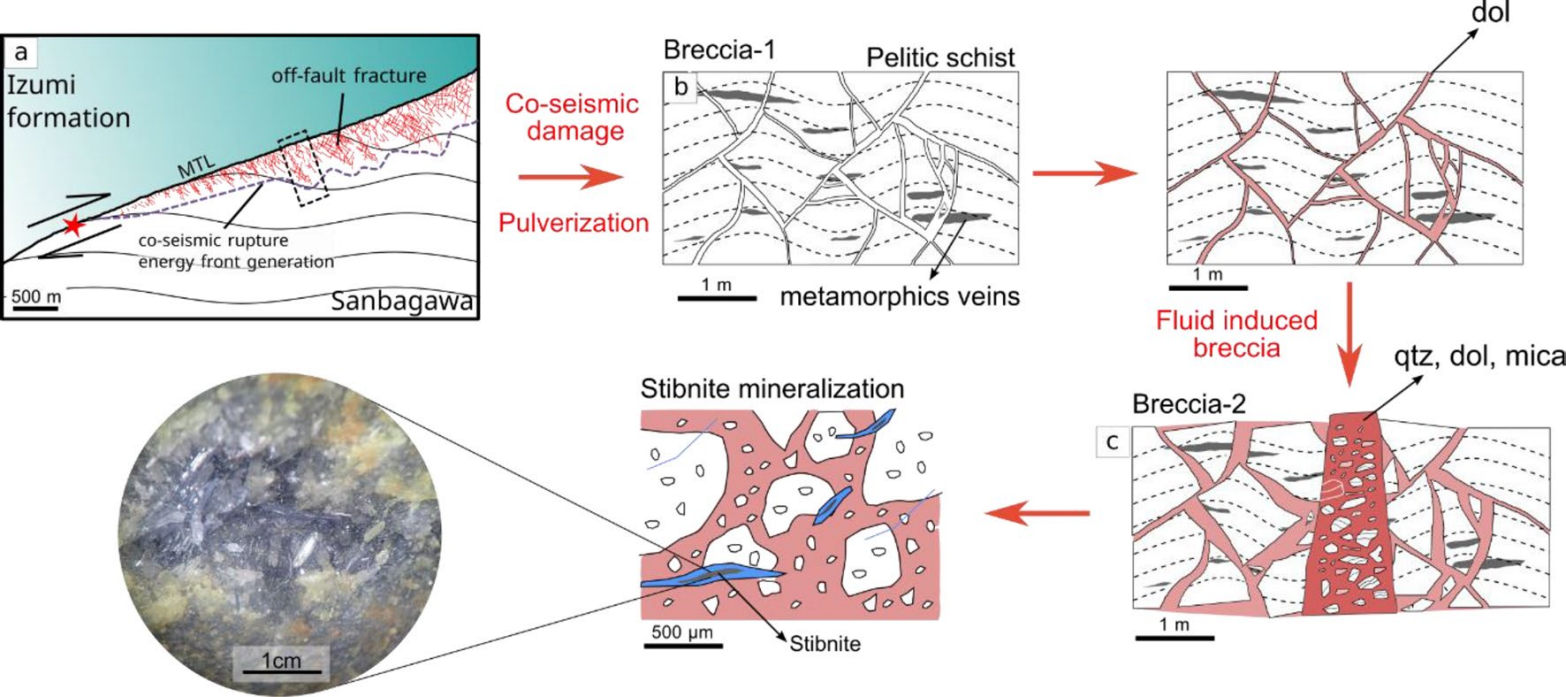
Brecciation model in Ichinokawa. (**a**) Schematic view of subshear rupture and energy propagation correspond to the activity along the MTL (**b**) Initial pulverization driven by rupture propagation during the coseismic event. (**c**) Fluid-induced brecciation as the consequence of elevated fluid pressure together with perfect macroscopic images of bipyramidal quartz and stibnite.

The previous investigation of breccia in Ichinokawa suggests that the rupture of pelitic schist with minimum rotation here is solely by hydro-fracturing without considering the contribution of seismic activity in the region^36^. Yet, we propose a model for the development of breccia based on the dynamics of fracturing in relation to the main tectonic fault and hydrothermal activity (Fig. 4) based on our novel investigation of regional geology, the internal structure of breccia, and multiscale PSD analysis. Elastic energy and surface energy (U_sa_) that are generated by the earthquake nucleation (red star in Fig. 4) along the MTL are responsible for the advancement of the rupture^6,18^. The bimaterial that is juxtaposed between Sanbagawa Formation and Izumi Group causes off-fault fracturing primarily in the stiffer rock, that is, pelitic schist^47,70^. Subshear rupture and pulverization propagate forward as the recurrence of the earthquake releases elastic energy, and these processes occur recursively^71^ (Fig. 4a,b). These processes caused the formation of bx-1, as rock pulverization occurred close to the MTL and dolomite filled microfractures during the initial brecciation (Fig. 4b). The formation of bx-1 increased the fracture permeability of pelitic schist leading to an episodic hydrothermal fluid flow that entrained the particle to form bx-2 as a breccia dike^11,45^ (Fig. 4c). Fluid pressure may be building up to some extent underneath Ichinokawa, even though the hydrothermal sources and activity remain uncertain^72^.

### Energy budget on off-fault damage through the multi-scale of breccia

**Figure 5:**
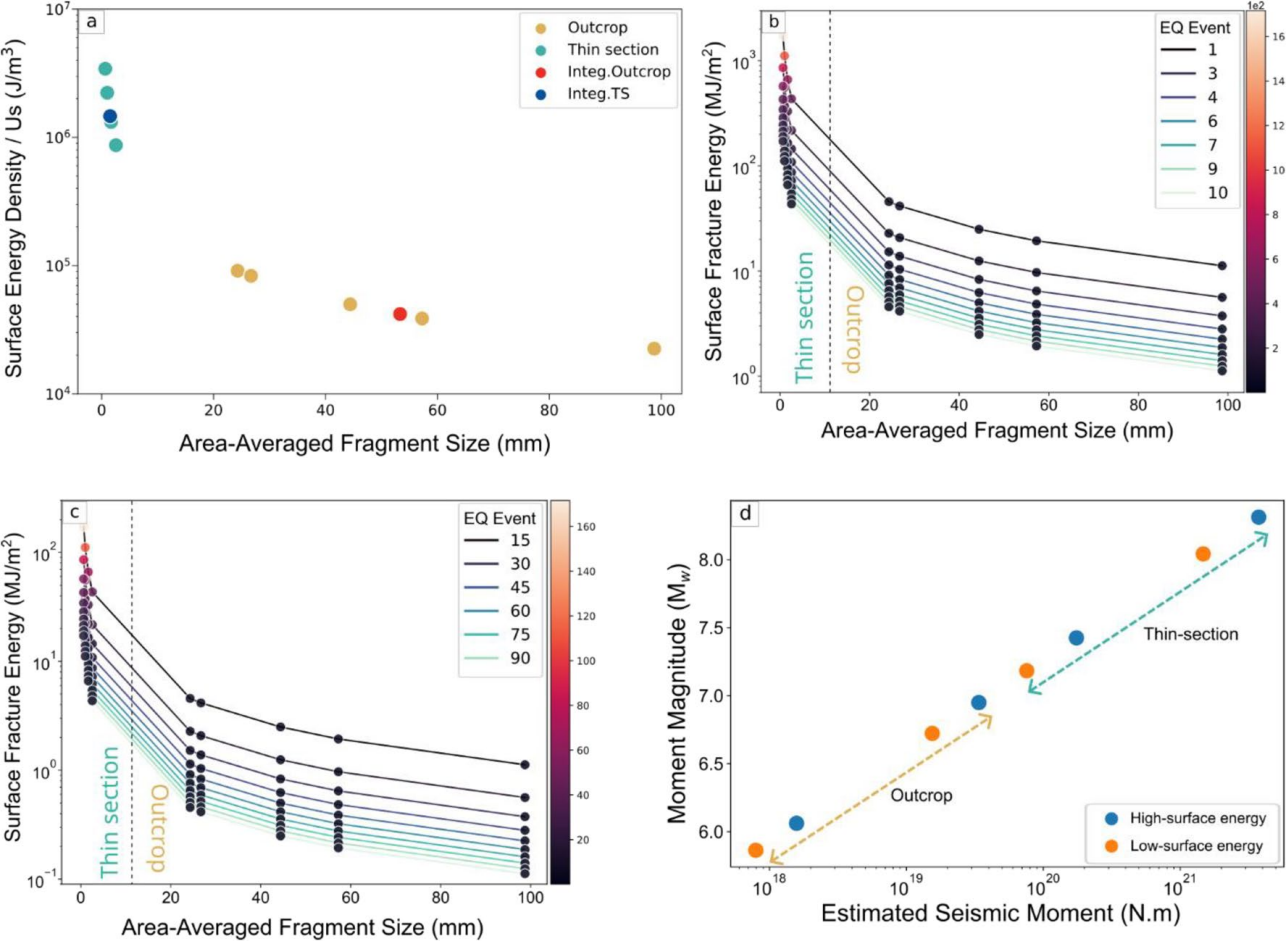
Surface energy calculation in Ichinokawa. (**a**) Surface area versus energy density in Ichinokawa based on outcrop and thin section (**b**) and (**c**) Estimation of surface energy per unit fault/damage zone for a single earthquake (U_sa_) assuming total earthquake recurrences of 10 and 100 to advance the rupture in Ichinokawa. (**d**) Calculated earthquake magnitudes according to the average surface energy for the 10 and 100 earthquake recurrence scenarios at the outcrop and thin-section scales.

Breccia that close to the main seismic fault experienced high coseismic damage. This pulverized rock is characterized by low shear strain and very high fracture intensity^34,73^. This texture is thought to be associated with paleoearthquakes and reflect the mechanical processes that occur during coseismic events^13,74,75^. The elastic energy is accumulate before the rupture and fracture energy can be dissipate to radiated energy and to surface energy during and after the slip event to advances the rupture of the intact rock^6,14^. We next estimated the energy budget based on off-fault damage using a D value of > 1.68 to perform calculations as the low fractal dimension might represent the low energy fragmentation, as explained in the previous section. Therefore, we assumed that fragmentation occurs in a single system (scale invariance). In our calculation we utilized both individual breccia data and integrated PSD data (Fig. 3a). Using those value and the method outlined by Johnson et al.^14^, we obtained the area-averaged particle size (L) for the outcrop at 24.30–8.73 mm and for the thin section at 0.65–2.55 mm. Assuming that the specific surface area (γ) of pelitic schist is equal to that of granite at approximately 56 J/m^2^^76–78^ and using surface correction for the most natural gouge of 6.6^28^, we calculated the total surface fracture energy density (*U*_*s*_) at 2.25–9.12 × 10^4^ J/m^3^ and 8.69 × 10^5^ to 3.43 × 10^6^ J/m^3^ for outcrop and thin sections, respectively. The scale-integrated data showed that the surface area and surface energy density are in the range of the energy indicated by individual breccia (indicated by red circle in Fig. 5a). It is evident that the fragmentation continues from the macro to micro scales, and the particle size dictates the energy consumption during the fragmentation (Fig. 5a and Supplementary Table 1). The estimated values of dissipated energy in Ichinokawa are comparable to those of several natural and experimental occurrences of off-fault fragmentation or pulverization^14,29,30,65^ (Supplementary Fig. 7a). The thin section scale records the highest energy dissipation. This is in line with dynamics fragmentation model by Grady, Glenn and Chudnovsky^79,80^, where the smallest aggregates are subjected to intense fragmentation processes at high strain rates.

The total energy density is distributed across the ~ 500 m (according to outcrop distance with respect to the MTL) wide dynamic damage zone to propagate the rupture through the off-fault region. Integrating the energy density (*U*_*s*_) over the damage-zone width in Ichinokawa resulted in the total fracture energy per unit fault area (*U*_*sa*_) of 1.12–4.56 × 10^7^ J/m^2^ for the outcrop and 4.34 × 10^8^ to 1.72 × 10^9^ J/m^2^ for the thin section. The pulverization accumulates the stress of every individual coseismic slip^65^. Thus, the estimation of surface energy for a single earthquake might reflects the dynamic loading during slip event that contributes to the fragmentation^81^. The total displacement of the principal fault can be used to estimate the number of earthquake events^20,21,28^. However, the estimation of earthquake recurrence based on the slip displacement cannot be deployed in Ichinokawa.

Therefore, we used the dolomite matrix as a proxy to obtain the number of earthquake events. Assuming dolomite cementation with apparent oscillatory zoning occurs during the coseismic event, we found five to eight zonings that reflect the minimum earthquake recurrences (Fig. 2g,h and Supplementary Fig. 1g). The estimation of surface energy released by a single earthquake in Ichinokawa is 1.12–172 MJ/m^2^ (Fig. 5b and Supplementary Table 1). An earlier study^22,28,34,64^ suggests that the pulverized rock observed near large seismic faults is a distinct type of rock fragmentation produced by intense dynamic loading. Therefore, we estimated the number of loadings subjected onto the intact rock to produce the finest breccia clast in Ichinokawa from the biggest fragment based on the dynamics loading experiment^82^. By fitting our grain size data to their experimental result we obtained approximately 100 loadings required to generate the finest clast size we observed in Ichinokawa; in this case, the calculated *U*_*sa*_ is 0.11–17.2 MJ/m^2^ (Fig. 5c and Supplementary Table 1). But it is important to note that calculated number of loadings based on grain size is straightforward without considering the processes during interseismic period like fracture healing and pre-existing fracture that could affected the loading geometry and stress/strain state during impact loading. This assumption could lead to less dynamics communition and underestimated the number of loading or earthquake cycle subjected to natural intact rock, subsequently reduce the surface energy estimation from natural fragmented rocks^71,82^.

The scale integration estimated the surface energy span at 2.08–73.13 and 0.21–7.31 MJ/m^2^ for 10 and 100 earthquake recurrences, respectively. This comparison shows that the energy budget is lower by four-fold compared with the average surface energy obtained from individual breccia data. This also influences the earthquake magnitude estimation.

These results are similar to those of other estimates of energy release for single earthquakes compiled by Johnson et al.^12^ (Supplementary Fig. 7b). Our energy estimation at the outcrop scale corresponds to those of large displacement faults such as Chelungpu, Punchbowl, and San Andreas Faults^20,21,73^, While at the thin-section scale, the surface energy in Ichinokawa is higher by two orders of difference compared to those large seismogenic zone. This is suggesting that the surface energy in Ichinokawa is stored across the wide damage zone of the MTL at the outcrop scale, and its significant quantity suggests that it is a non-negligible component for the advancement of the rupture near the principal fault zone. The discrepancies between observational scale suggest that the highest energy is dissipated and recorded at the thin section scale and estimation of energy at this scale potentially to be overestimated. Additionally, a width of 500 m of the dynamical damage zone corresponds to a depth of ~ 5 km according to the physic-based dynamics earthquake model provided by Okubo et al.^6^. The corresponding depth was confirmed by estimating the pressure based on the isochore analysis of fluid inclusions at 30–190 MPa (Supplementary Fig. 8 and detailed explanation to obtain the number is in Supplementary text). According to the model at this depth, approximately 40% of the fracture energy is dissipated in the off-fault medium, and the rest of the energy is directed into the fault^6^. Which indicates that energy partitioning in the off-fault region cannot be neglected.

### Estimation of earthquake magnitude and comparison with slip distribution for paleo and recent earthquake events on MTL in Shikoku

Given the surface energy for every earthquake event, we estimate the seismic moment (*Mo*) and moment magnitude (*Mw*) that operated during the brecciation. For the outcrop, the estimated *Mo* ranges from 7.98 × 10^17^ to 3.38 × 10^19^ corresponding to *Mw* values between 5.8 and 6.9. Whereas, at thin section scales the *Mo* gives a value of 7.57 × 10^19^–3.74 × 10^21^ and corresponds to *Mw* of 7.1–8.3 (Fig. 5d and Supplementary Table 1).

**Figure 6:**
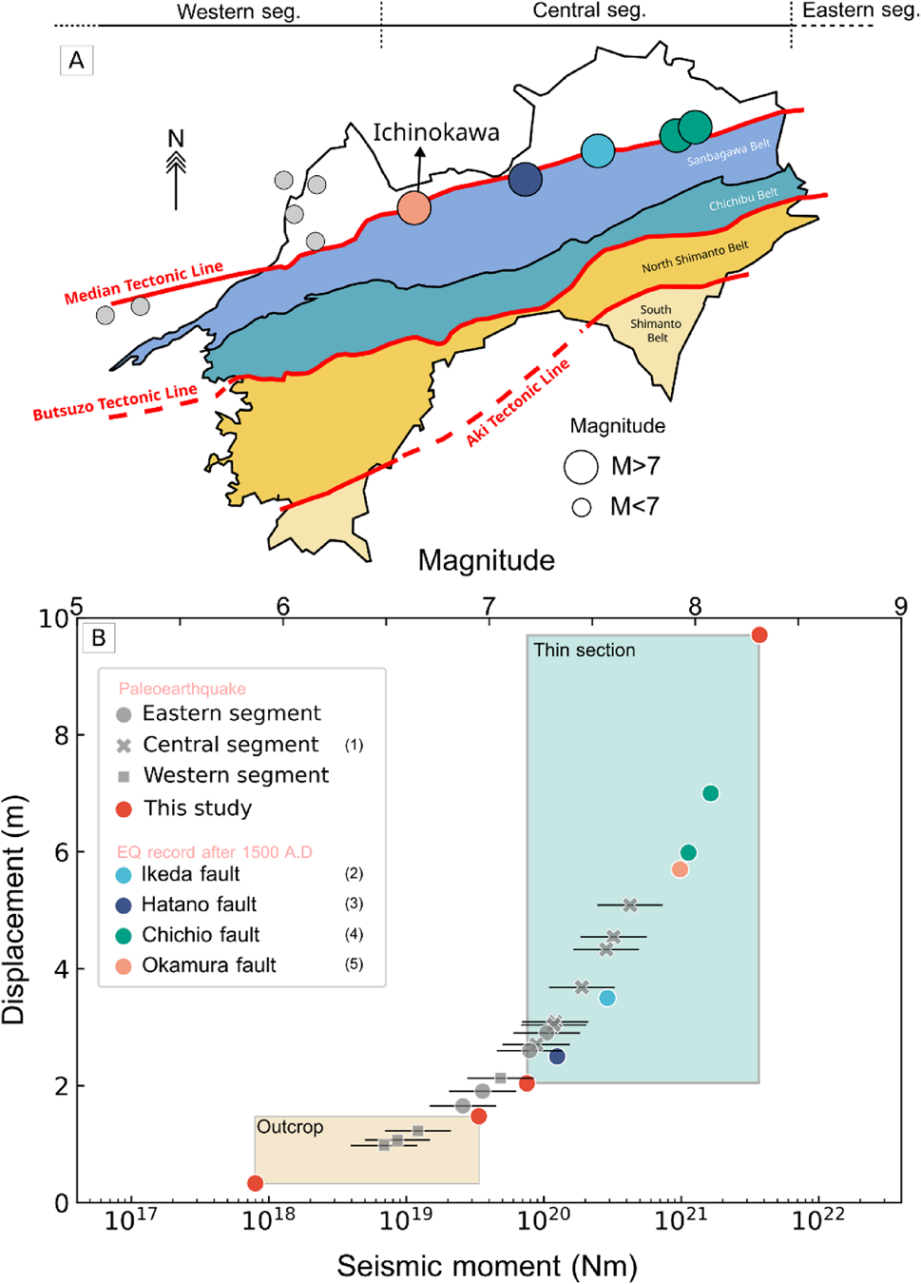
Slip displacement of earthquake records around MTL is compared to the seismic moment and magnitude data. (**A**) The distribution of all recorded earthquakes from paleo to recent events, adjacent to MTL in Shikoku Island is depicted. (**B**) The graph shows the paleoearthquake data estimated from morphological features around MTL for each fault segment, with bars representing estimated earthquake magnitude based on average event recurrences in MTL, which is 1000–3000 years^39^. The data for paleo and recent earthquake events are compiled from following sources: (1) Kanaori et al.^42^; (2) Patria et al.^83^; (3) Goto et al.^84^; (4) Okada and Tsutsumi^85^; (5) Tsutsumi^39,44^.

The central segment, which is the longest portion of the MTL, plays an important role as most earthquakes greater than magnitude 7 are recorded here (Fig. 6A). Tsutsumi et al.^44^ reported the latest seismic events recorded on Okamura segments that coincided with Ichinokawa was happened during 8th A.D. with displacement 5.7 m, corresponding to a magnitude of 7.9. Some documentation on recent activity (> 1500 A.D.) of MTL from several segments show that the layer offset of Quaternary sediment is at 3.5 m, 6–7 m, and 2.5 m for Ikeda, Chichio, and Hataro fault segments respectively^83–85^. These values can be translated into magnitude of 7.57 for Ikeda fault, ~ 8 for Chichio fault, and 7.3 for Hatano fault (Fig. 6B). Considering the characteristic of slip rate for all segment in ranges 0.5–9 mm/yr^42^ with a recurrence interval of surface-rupturing earthquakes of 1000–3000 years^39^, the minimum average slip is 1 m to 7 m for an earthquake on the MTL in Shikoku, corresponding to magnitudes of 6.5–7.7 (Fig. 6B).

According to all documented earthquake magnitudes in both paleo and recent coseismic activity within the region, it is evident that earthquake estimation from surface energy falls within the range of those values and is comparable to typical coseismic intensity and activity on the MTL, especially in the central segment. It is suggested that the main fault activity can be approximated through the measurement of breccia in the off-fault region. Moreover, we postulate that we can extrapolate this novel approach to other seismogenic zones because we have shown that the surface energy is comparable with other regions and is proportional to seismic moment and earthquake magnitude. In addition to that, we also realize that many mechanical and physical parameters need to be constrained to estimate the surface energy and earthquake magnitude, including the spatial heterogeneity of fault properties, fracture density, and type of converted energy, which can be the source of error/uncertainties and affect the result of seismic moment estimation. So it should be interpreted with appropriate care and in the future, we should accumulate a significant amount of data to generate a robust surface energy to seismic moment model.

## Methods

### Electron microprobe analysis

The mineral compositions of the samples were determined using an electron microprobe analyzer (EPMA; JEOL JXA-8200) at Tohoku University, Japan. with the accelerating voltage, beam current, and beam diameter of 15 kV, 12 nA, and 1 μm, respectively. Natural and synthetic standards were used to calibrate the instrument (wollastonite for Ca and Si, rutile for Ti, eskolaite for Cr, hematite for Fe, manganosite for Mn, periclase for Mg, albite for Na, feldspar for K). For each major element, the counting times deployed for peaks and backgrounds were 10 and 5 s, respectively. For X-ray mapping using EPMA was performed with acceleration voltage, beam current, and dwell time of 15 kV, 120 nA, and 60 ms, respectively. The beam diameter was 1 µm.

### Scanning electron microscopy coupled with cathodoluminescence

The internal quartz structure was characterized using a scanning electron microscope equipped with an Oxford cathodoluminescence detector and photomultiplier (SEM-CL) at the Graduate School of Science, Tohoku University, Japan with an accelerating voltage of 25 kV and a beam current of 90 µA. The CL image was analyzed as described by Frelinger et al. and Rusk and Reed^86,87^.

### Fluid inclusion microthermometry

Fluid inclusion microthermometry was performed on double-polished thick sections (~ 100-µm thickness) of the Sb-bearing vein to evaluate the fluid temperature during mineralization and the thermal properties at the onset of brecciation particularly breccia type 2. Microthermometry was performed using a Linkam THS600 heating/freezing with an operating temperature of  − 180–600 °C and a measurement error of 0.1 °C at the Tohoku University, Japan. Salinities of fluid inclusion in NaCl wt.% equivalent were estimated using the final melting temperatures of ice^88,89^.

### Raman

Raman spectroscopic analysis was applied to identify the phases of fluid inclusion from the Sb-bearing vein. Fluid type then compared with matrix assemblage in breccia to show whether the remnant fluid from brecciation processes is trapped and influences the mineralization processes. This work was done by using a HORIBA XploRA PLUS Confocal Raman Microscope at the Graduate School of Environmental Studies, Tohoku University using a 532 nm green laser. The laser power was 2–10 mW on the sample surface focused using 100 × magnification lens. Most spectra were collected using a 1800 grooves mm^−1^ grating. The Raman peak position was calibrated using a silicon wafer (520.7 cm^−1^).

### PSD at macro- and microscales

PSD was used to understand the characteristics and formation mechanism of breccia in Ichinokawa. Scanline sampling was deployed both for direct field and rock slab/thin-section measurements to characterize the matrix-to-clast ratio (ε) and the distribution of clast within the breccia. The one-dimensional scanline was conducted to obtain the geometry of the breccia or clast parameter primarily in the long (L) and short (S) axes of the clast/particle for breccia-2 (bx-2; Supplementary Fig. 6c,d). The PSD measurement is a function of the width of breccia pipe. For the rock slab/thin-section, we initially traced the image that fits the sampling line, and then, obtained the clast parameters using ImageJ (Supplementary Fig. 6e,f)^90^.

The clast size is provided by the square root of L and S of each particle, and it is expressed as $d={(LS)}^{1/2}$. The cumulative probability was calculated from the PSD^66^ and then plotted in the log(N) − log(d) diagram. The distributions were fitted using the power law equation as follows:

1 $$N \propto d^{ - Ds} .$$

where d and N denote the particle size (µm) and the cumulative probability of particles > d, respectively, and Ds is the fractal dimension^91,92^. Furthermore, the multiscale particle size integration was performed by normalizing the clast distribution with the measurement area (mm^2^) to obtain the particle density as function of clast size.

### Calculation of surface energy and earthquake magnitude

For the estimation of the surface energy using natural fragment size data, we assume the grain size reduction mostly govern by co-seismic event. The small contribution derived from fluid wearing is being neglected due to the absence of observed post-deformational fragmentation processes like grain healing-fracturing and clast alteration. The grain size transformed the mean diameter into the area-averaged fragment size. The versatile derivation of this problem was performed according to Johnson et al.^14^ by considering the surface area per unit volume of a fragment consisting of *n*_*i*_ spheres of diameter *s*_*i*_ for bin *i* as follows:

2 $$\left(\frac{A}{V}\right)=\frac{\Sigma {n}_{i}\left[4\pi {\left(\frac{{s}_{i}}{2}\right)}^{2}\right]}{\Sigma {n}_{i}\left[\frac{4}{3}\pi {\left(\frac{{s}_{i}}{2}\right)}^{3}\right]}=\frac{6\Sigma {n}_{i}{s}_{i}^{2}}{\Sigma {n}_{i}{s}_{i}^{3}}=\frac{6}{{\stackrel{`}{s}}_{avg}}=\frac{6}{L}$$

For surface area (L) the Eq. (2) can be rewritten as follows:

3 $$L=\frac{\Sigma {n}_{i}{s}_{i}^{3}}{\Sigma {n}_{i}{s}_{i}^{2}}$$

The count of the fraction for the power-law cumulative distribution of a population of spheres (*n*) can be expressed as follows:

4 $$n\left(s\right)={kDs}^{-D-1}$$

where *s* is the diameter of the fragment, *D* is the fractal dimension, and *k* is the constant. By integrating Eqs. (2 and 4), we can obtain:

5 $$L=\frac{\Sigma {n}_{i}{s}_{i}^{3}}{\Sigma {n}_{i}{s}_{i}^{2}}=\frac{{\int }_{{s}_{min}}^{{s}_{max}}n\left(s\right){s}^{3}ds}{{\int }_{{s}_{min}}^{{s}_{max}}n\left(s\right){s}^{2}ds}=\frac{{\int }_{{s}_{min}}^{{s}_{max}}{kDs}^{-D+2}ds}{{\int }_{{s}_{min}}^{{s}_{max}}{kDs}^{-D+1}ds}$$

Thus, the solution is:

6 $$L=\left(\frac{2-D}{3-D}\right)\left[\frac{{s}_{max}^{\left(3-D\right)}-{s}_{min}^{\left(3-D\right)}}{{s}_{max}^{\left(2-D\right)}-{s}_{min}^{\left(2-D\right)}}\right]$$

The area-averaged fragment size (*L*) as a function of diameter (*s*) and fractal dimension (*D*) allows us to estimate the surface energy density (*U*_*s*_; J/m^3^) using the following equation:

7 $$Us=\frac{6\gamma \lambda }{L}$$

where γ is specific surface energy of the material (J/m^2^) and λ represents the surface-area correction factor because the breccia fragment is not a perfect sphere or cube.

Using the converted energy value from the numerical and kinematic slip model coupled with the seismic moment (*M*_*o*_) allows us to generate a simple model of the linear relationship between energy and seismic moment (Supplementary Fig. 4c,d). Assuming that the energy generated during the earthquake is converted into surface-area energy (*U*_*sa*_), we fitted the average energy value of two earthquake recurrences in Ichinokawa (10 and 100 EQ) to get the seismic moment. Subsequently the moment magnitude (*M*_*w*_) was estimated using the equation provided by Kanamori and Brodsky^37^ as follows:

8 $$Mw=\frac{log\left(Mo\right)}{1.5}-6.07$$

For the case of Japan inland surface faulting, given the magnitude can be translated into displacement of major fault that induced brecciation by using following equation^93^:

9 $$\text{log}(D)=0.6M-4.0$$

where D is displacement in meter and M is earthquake magnitude.

### Supplementary Information

Supplementary Information.

## Data Availability

The datasets generated and analyzed during the current study are available from the corresponding author on reasonable request.
