# Supplementary material for: Multiscale off-fault brecciation records coseismic energy budget of principal fault zone
Source: Sci Rep. 2024 May 27;14:12121. doi: 10.1038/s41598-024-62838-x (PMC11130164; doi:10.1038/s41598-024-62838-x)
Supplement: Supplementary file 1 — Supplementary Information. [file 41598_2024_62838_MOESM1_ESM.docx]

***Supplementary Information***

**Multiscale off-fault brecciation records coseismic energy budget of principal fault zone**

Geri Agroli^1^, Atsushi Okamoto^1^, Masaoki Uno^1^, Noriyoshi Tsuchiya^1,2^*

1. Graduated School of Environmental Studies, Tohoku University, Sendai 980-8579, Japan

2. National Institute of Technology, Hachinohe College, Hachinohe, Aomori 039-1192, Japan

* Corresponding author:

Noriyoshi Tsuchiya

*noriyoshi.tsuchiya.e6@tohoku.ac.jp*

This PDF file includes:

Supplementary text

Supplementary Figures 1 to 8

Supplementary Tables 1 to 4

# Supplementary Text

## **Temperature estimation and composition of ore-forming fluid**

Arsenopyrite is quite ubiquitous and it corresponds with antimony mineralization and paragenetically considered as the early stage of mineral deposition ^6,7^. The composition of arsenopyrite can be utilized in buffered assemblages as a cationic geothermometer following ^8^ and later modified by ^9^ . In Ichinokawa euhedral-arsenopyrite and pyrite disseminated on the matrix of the bx-2. The core-to-rim measurements show that arsenopyrite’s chemical composition spans from 26 to 30 atomic percent of arsenic (As) (At%). Assuming that the arsenopyrites are at equilibrium with pyrite as buffered assemblages, the calculated temperatures are in the range of <300°C to 360°C (Supplementary Fig. 6a-c and Table. 2). We consider the onset of breccia-2 (bx-2) formation to be around that temperature and it influences the physicochemical condition of the fluid afterward. An attempt to measure the titanium content in quartz-matrix was also conducted, however, no titanium was detected indicating that this particular quartz precipitate at a temperature under 514°C corresponds to titanium concentration less than 7ppm ^10,11^, and It is consistent with our arsenopyrite geothermometer.

Fluid inclusion (FI) was performed on euhedral quartz and barely from syntaxial quartz of the Sb- bearing vein (Supplementary Fig. 5d). The measurement tries to encompass the multiple quartz generation as we mentioned above, yet the inclusion size <2 µm on average makes it more challenging to tie CL-image and FI therefore, we assess the inclusion according to the relative position of core or rim. The FI has elongated and sub-rounded assemblages consisting of vapor and liquid phases of H_2_O (Supplementary Fig. 5e).

Based on Raman analysis, the liquid and vapor have broad peak intensities of 3100-3600 cm^−1^ corresponding to water. The vapor contains a small amount of CO_2_, this is also suggested petrographically by the presence of darker color at the rim of the bubble (Supplementary Fig. 5f). According to the microthermometry analysis, the homogenization temperature (Th) of core-area is span around 221 to 325°C and the rim side is in the range of 115-183°C. A limited amount of ice melting temperature (Tm) was obtained yielding the temperature of -5.7 to -1.2°C which corresponds to salinity ∼2 to 8.8wt% (NaCl eq.) respectively (Supplementary Fig. 5g and Table. 3).

## **Characteristics of stibnite mineralization in Ichinokawa**

The occurrence of Ichinokawa breccia has been subjected to a long history of stibnite mines. Visible stibnite is mainly found near the mine area either the adit or mine tail, here pelitic schist or breccia with size boulder to pebbles contains stibnite vein. Unfortunately, we can’t encounter an Sb-related outcrop. In our three sampling points, stibnite most likely occurs in two mineralization styles. First is stibnite deposited parallel with schistosity or lineation of pelitic schist accompanied by the presence of yellowish mineral and relatively porous with a vivid void spread through the sample (Supplementary Fig. 6a). The second occurrence and the most common one is stibnite presents as a veinlet within the breccia this veinlet truncates both breccia (bx1 and bx-2) and intact pelitic schist, and later we consider stibnite as the last product of fluid evolution in Ichinokawa (Supplementary Fig. 6b).

The more details observation of stibnite veinlet indicates that stibnite deposition is accompanied by occurrences of needle-quartz crystals or bipyramidal quartz ^1,2^. Quartz crystals grow in two different styles (Supplementary Fig. 6c). The initial quartz grows as syntaxial quartz from the vein wall (as substrate) to the center of the vein and perfect individual doubly terminated crystal (euhedral) quartz floating in the surrounding in close association with stibnite crystals appears as a filling of veinlets and cavities, postdating arsenopyrite. Cathodoluminescence shows that syntaxial quartz has a low luminosity (CL-Dark) with no zoning, in contrast, the euhedral quartz has three distinct textures representing different quartz generations from core to rim (Supplementary Fig. 5d) (the schematic figure). The earliest stage (qtz-a) has bright luminescence with zoning and dissolution texture observed in the rim part. The second stage (qtz-b) has a CL-Gray texture with less zoning area compared to qtz-a, they often occur as inclusion within the core of qtz-a. And the last stages (qtz-c) show the lowest CL intensity with clear oscillatory zoning, they evenly intruded through prior deposited quartz (qtz-a and qtz-b). The plausible fluctuation of P-T of the fluid can lead to abrupt dissolution and precipitation of quartz under hydrothermal conditions ^3–5^.

## **Mechanism and source of ore-forming fluid for the massive mineralization of stibnite**

One of the highlights at Ichinokawa is how to explain the formation of massive stibnite crystals. Stibnite (Sb) occurs in a wide variety of geological settings. Magmatic-related processes are a common antimony deposit that hosts most stibnite including the other ore-mineral. Sedimentary antimony deposit occurs as strata-bound deposit and antimony-enriched in coal seams. The structure-related antimony is a less-extend deposit that hosted two typical stibnite mineralization with distinct mineral assemblages. First is the Monotonous Sb deposit consisting of monomineralic-monomineralic Sb within the vein. It occurs within the shear zone of non-metamorphic to low-grade metamorphic rock at low temperatures. It corresponds to a relatively distal heat source compared to the second structural-related deposit of polymetallic Sb-(Au-W-Sn-As-Zn-Pb) type ^12^.

The formation of structural-related antimony involves orogenesis and its dynamic process. The fluid derives from metamorphic dehydration percolate through structural features during regional deformation and subsequently mixes with the superficial fluid in the upper crust. Stibnite mineralization in Ichinokawa consists of monomineralic Sb (with some sb sulfosalt) which is present as veinlet and strata bound parallel to schistosity. The deposition of stibnite postdates the formation of breccia-2 because the vast amount of veinlet truncates both breccia-2 and pelitic-schist in the sample. The high concentration of Sb corresponds to breccia-2 also reported by ^13^. Syntaxial and euhedral quartz followed by the precipitation of stibnite as void-filling minerals (Supplementary Fig. 6c). Such kind of texture and characteristics are typical for shallow sb deposits associated with orogenic and hydrothermal processes exemplified in Armorican Massif, France, and West Turkey ^6,14^ where in Ichinokawa this processes perhaps coeval with late stage of Sanbagawa exhumation processes ^15^ or there is a hidden magmatic-hydrothermal system underneath Ichinokawa ^13^.

The stibnite deposition is occurs at low temperature ~80-220°C ^16,17^. The temperature derive from arsenopyrite suggest that the stibnite is remain soluble ^18^ and the onset of stibnite deposition is coincide with quartz temperature (Supplementary Fig.5i). The formation of massive stibnite mineralization requires dramatic changes of fluid condition such as P-T-X-*f*-pH ^19–21^. The pressure of 0.16–11.47 Mpa estimated from fluid inclusion ^22^ (Supplementary Fig.6h). The result is comparable with fluid boiling from several outstanding stibnite deposit like French massif and Xikuangshan ^7,23^. The pressure fluctuation of ore-forming fluid during stibnite deposition could lead to subsequent fluid cooling by decompression ^21,24^. Perfect euhedral texture or bi-pyramidal quartz can be an indication of decompression processes by flashing events leading the instantaneous and efficient mineral precipitation (Supplementary Fig. 6d) ^25,26^. Moreover, the decrease in the sulfur fugacity (depleted sulfur on the rim of pyrite and arsenopyrite) in low Ph condition (vuggy texture) also enhanced the stibnite scavenging from ore-forming fluid (Supplementary Fig. 2).

# Supplementary Figures


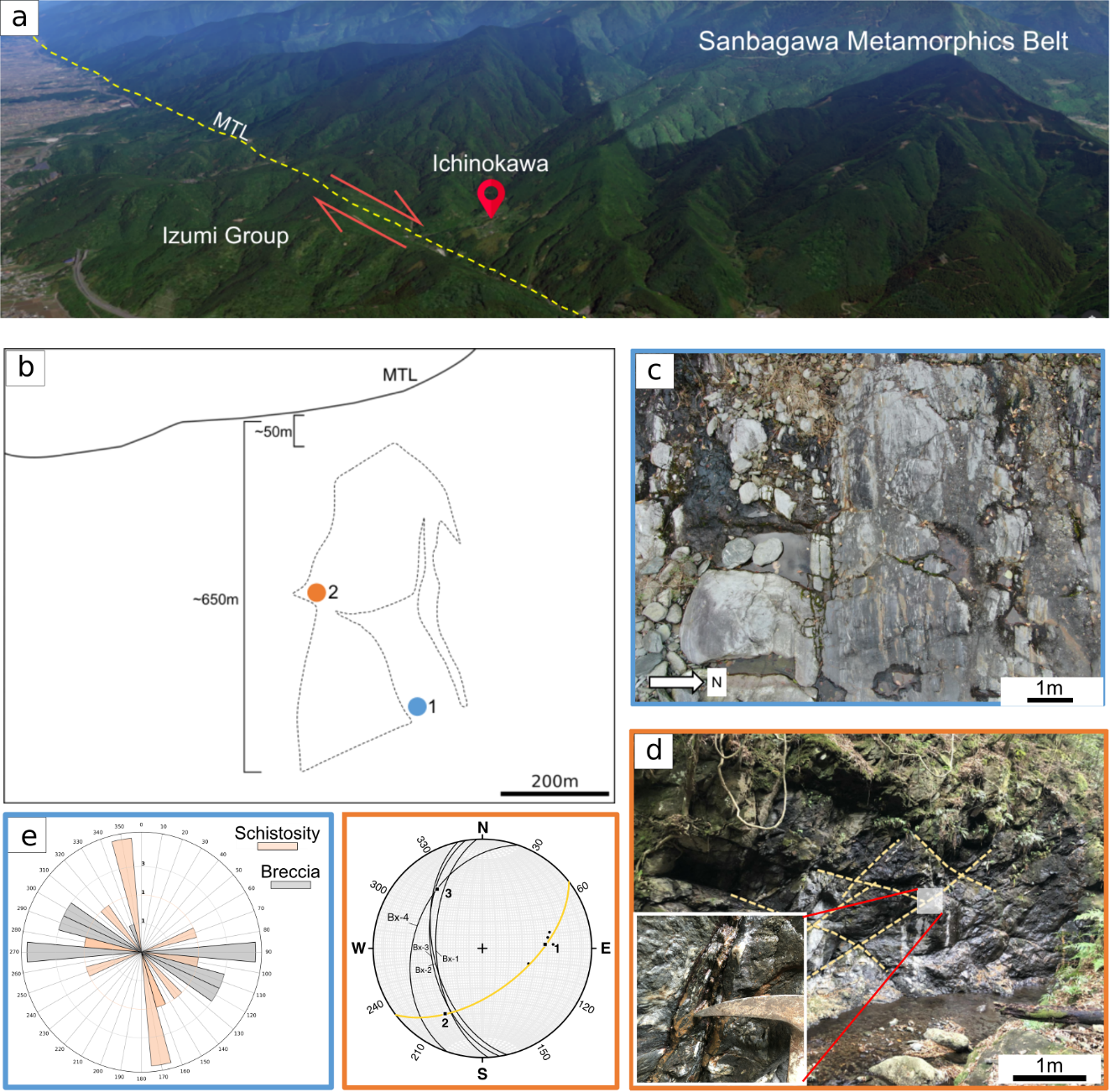


Supplementary Figures 1. **Ichinokawa and its relationship with MTL** (a) Ichinokawa is very near to median tectonic line (MTL) and the breccia is more likely controlled by the movement of MTL. (b) Simplified outline of MTL and local fault in Ichinokawa with two observation points. The distance between Ichinokawa and MTL is relatively close with the dynamics-wide of damage zone of observed outcrop ~500m. (c) At location 1 (marked in blue) the orientation of the breccia and fault parallels the MTL. (d) While at location 2 (orange) the orientation of the conjugate faults tends to be perpendicular to the MTL. (e) This implies that dynamics rupture occurs in this area according to kinematics analysis.


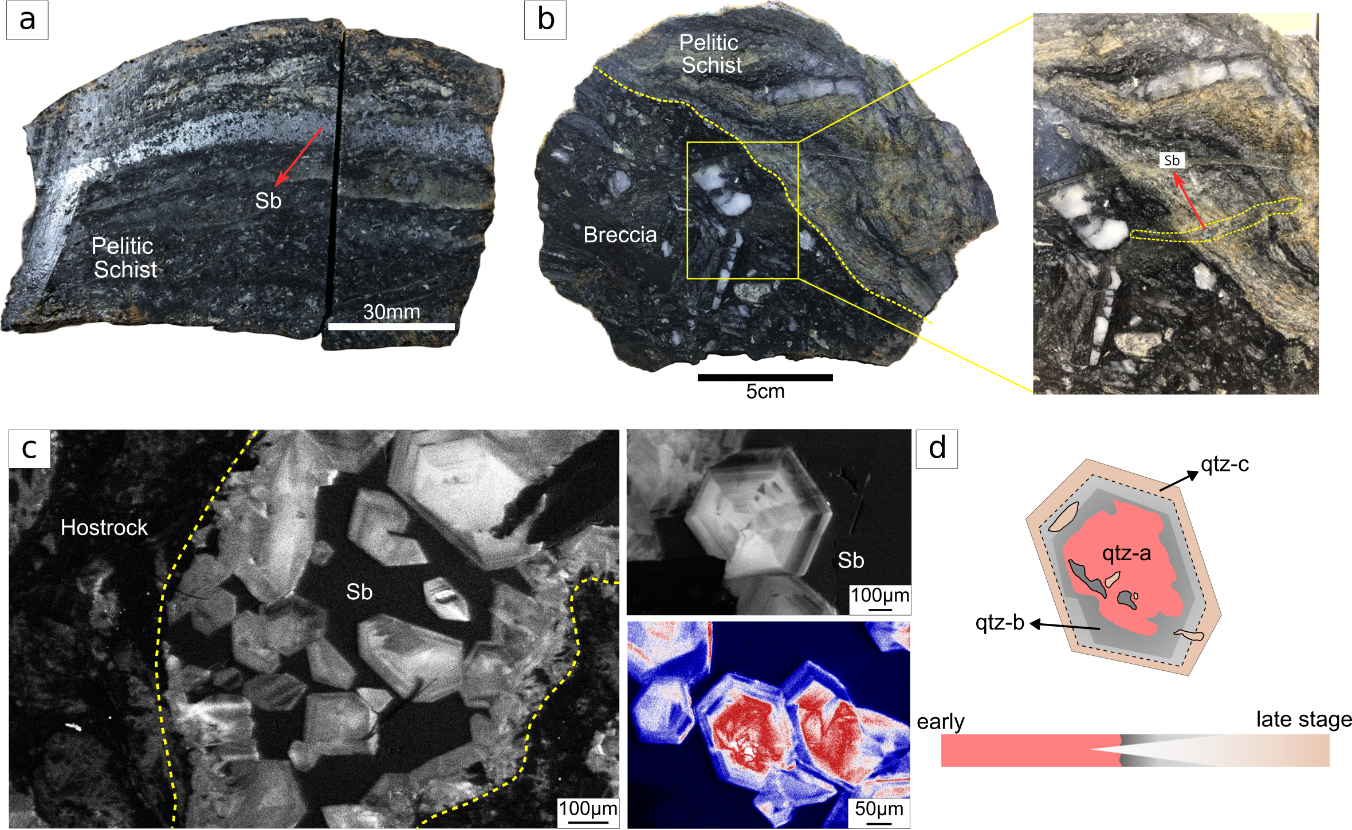


Supplementary Figures 2. **Mineralization style of Stibnite in Ichinokawa.** (a) Stibnite disseminated within the pelitic schist and as a vein. (b) Stibnite occurs as a veinlet and truncates both breccia and pelitic schist. (c-d) SEM-CL of stibnite vein shows the perfect euhedral quartz with successive zoning and denotes the evolution of the fluid throughout the vein generation.


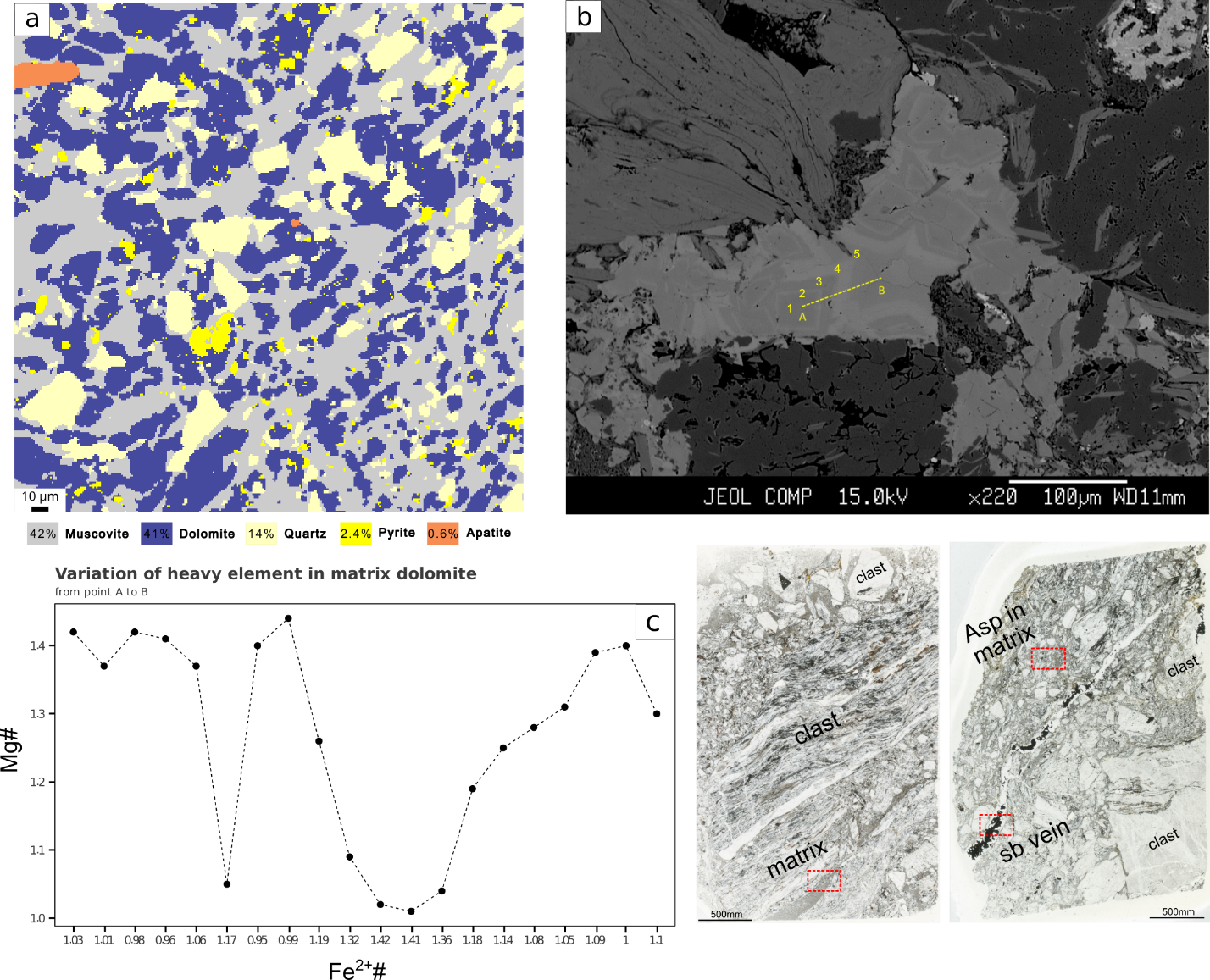


Supplementary Figures 3. **The matrix component and dolomite composition** (f) Relative abundance of the matrix. (a-c) The dolomite profile within the matrix shows the variation of Mg and Fe corresponding to five oscillatory zoning under backscattered image (BSE). A thin-section scan with a red-dashed square indicates the areas analyzed for a couple of analyses in this study.


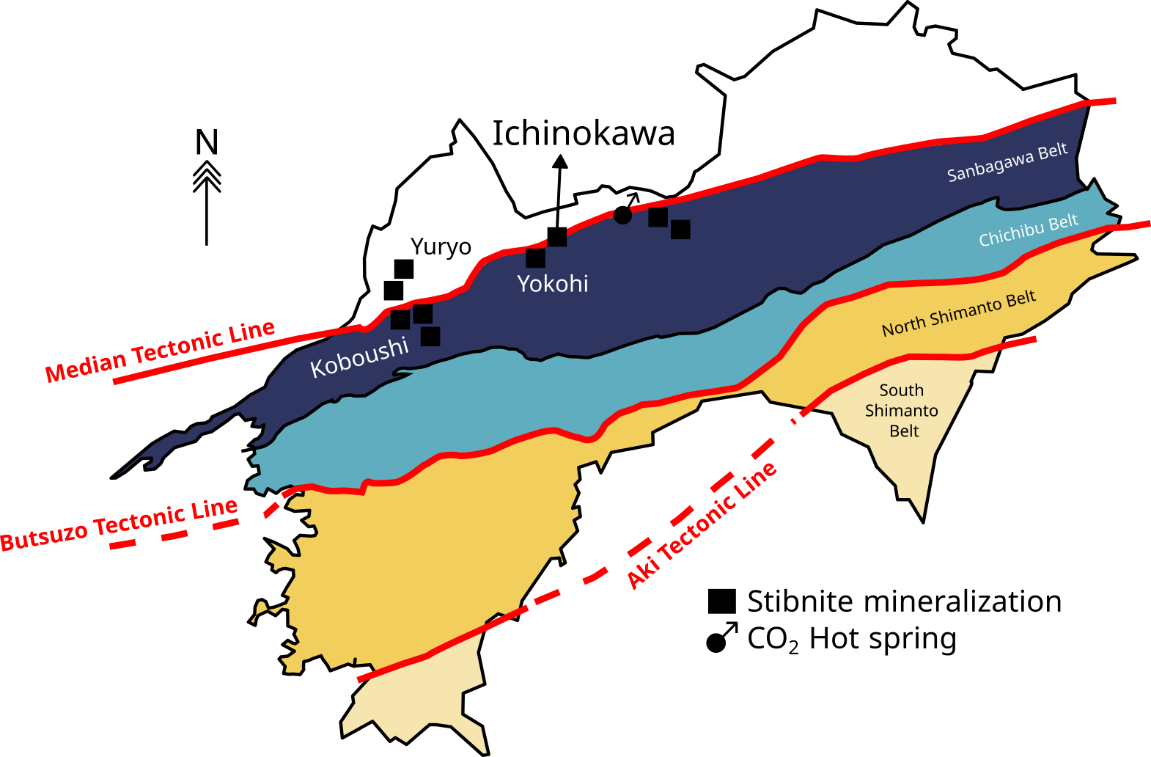


Supplementary Figures 4. The distribution of stibnite mineralization and CO_2_ hot spring show fluid activity is strongly associated with MTL.


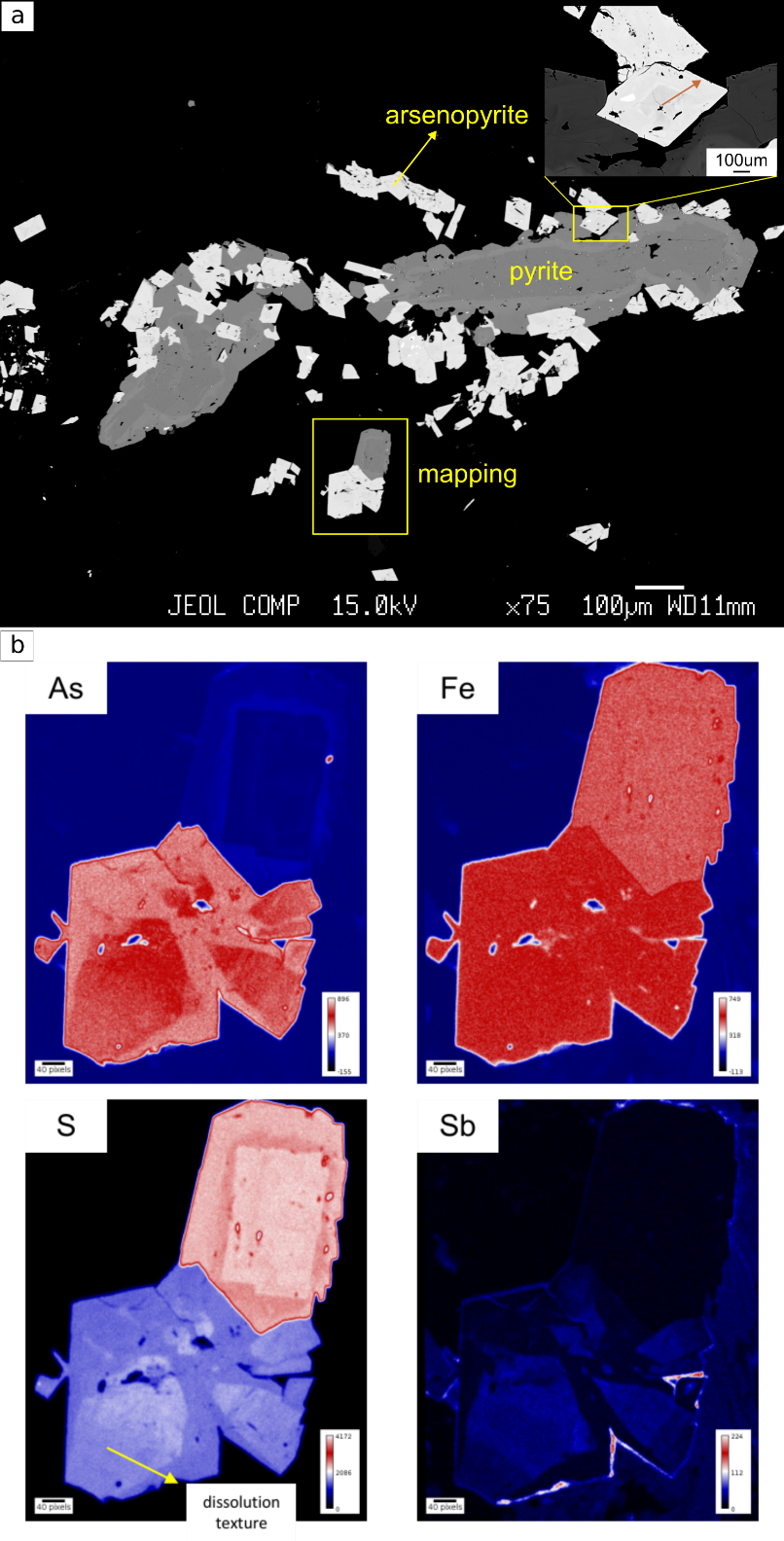


Supplementary Figures 5. **Texture, composition of arsenopyrite** (a) Breccia matrix with an abundance of sulfide minerals is commonly pyrite and chalcopyrite. (b) Elemental mapping on each sulfide mineral shows changes in composition mainly arsenic and sulfur accompanied by local dissolution textures.


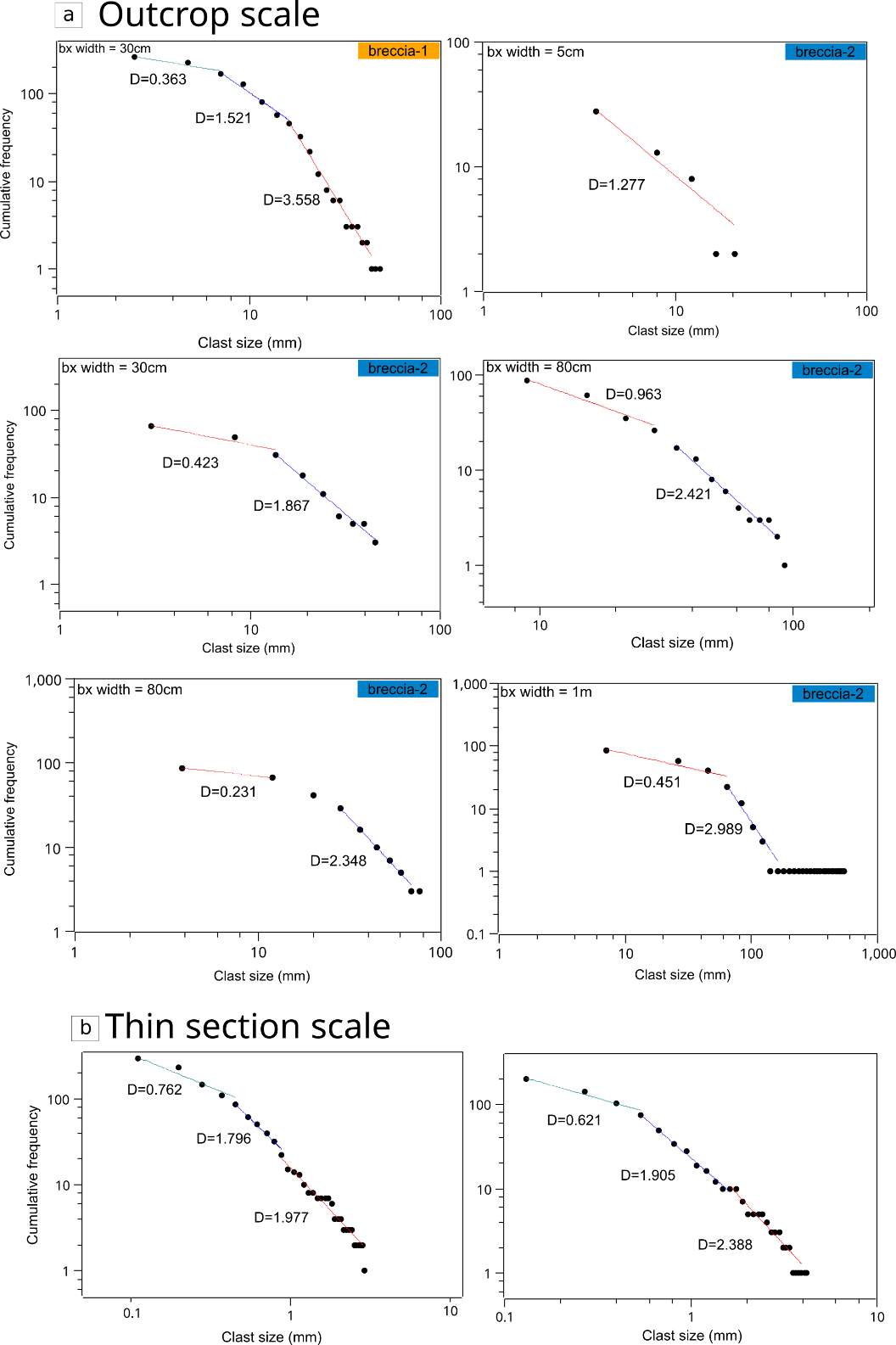


Supplementary Figures 6. **Particle size distribution (PSD) result.** (a-b) The result of PSD with fractal dimension (D value) from various breccia types and width on an outcrop and thin section scale.


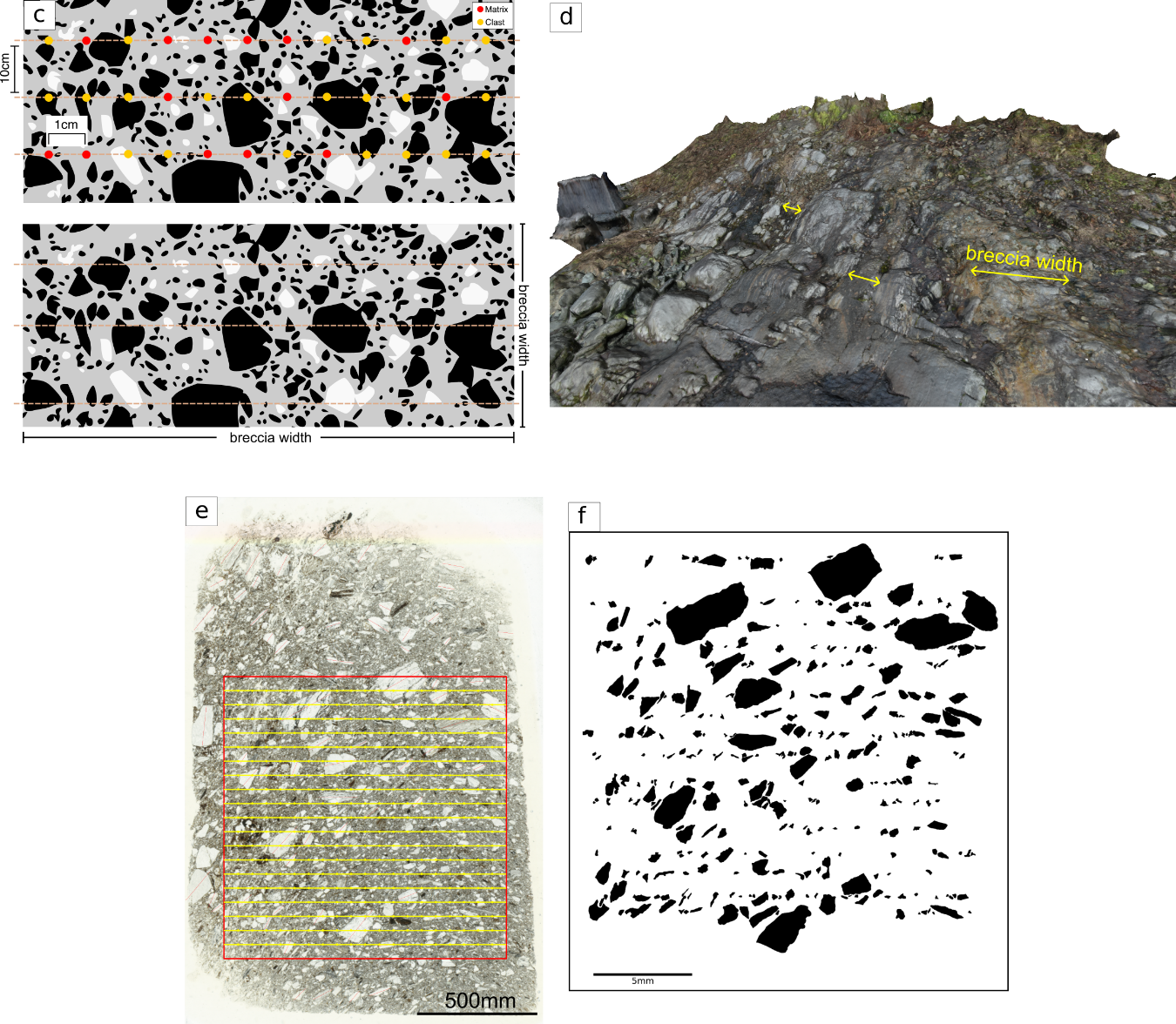


Supplementary Figures 6. **(continue)** **Method to obtain Particle size distribution (PSD) of breccia.** (c) Scanline method was deployed to obtain breccia clast parameter like long and short axis on (d) an outstanding breccia exposure in Ichinokawa. (e) Scan of whole thin section and (f) manual sketch of breccia clasts used for obtaining the particle distribution of the clasts.


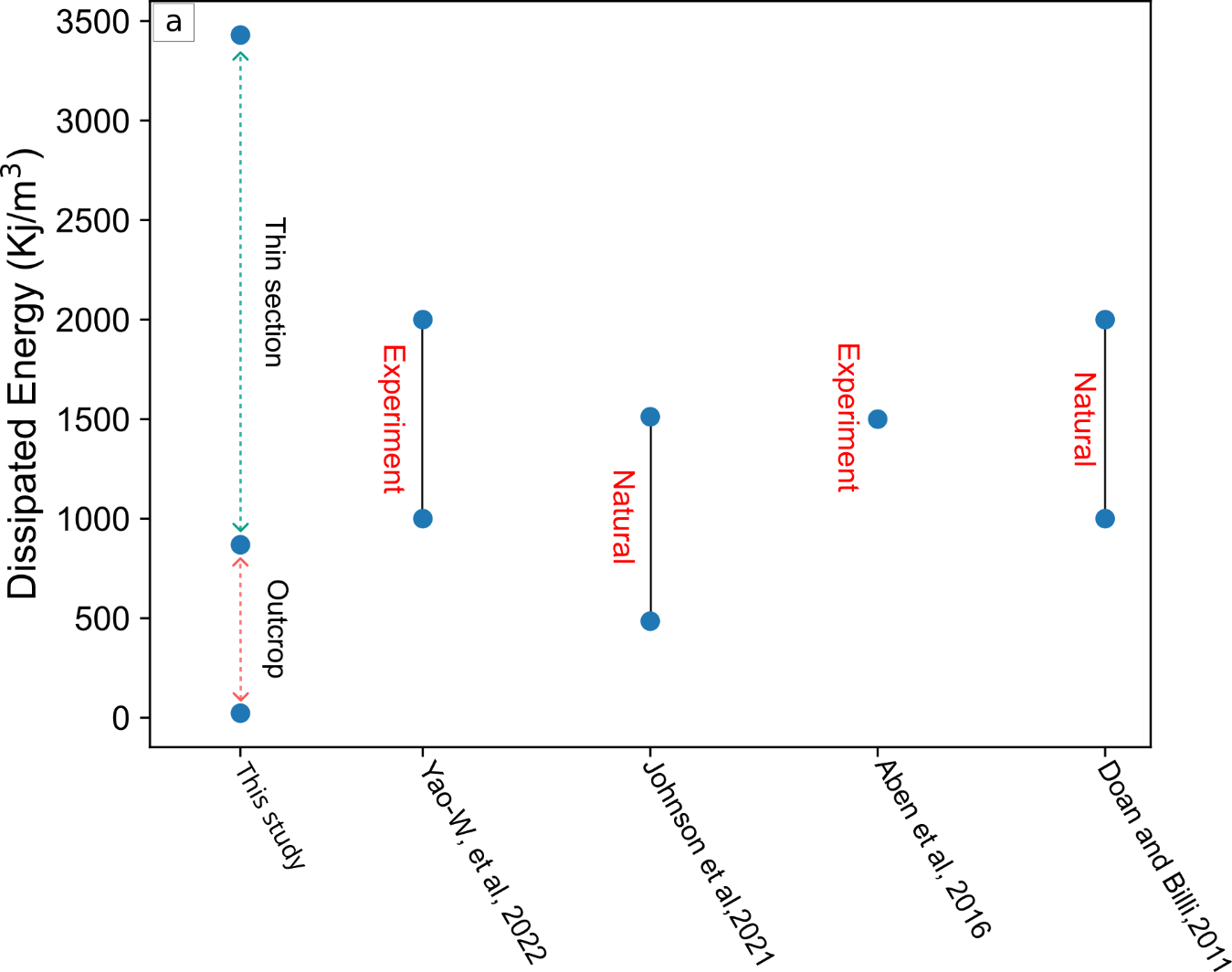


Supplementary Figures 7. **Plot of total surface energy density (U_s_)** (a) Comparison of calculated U_s_ in Ichinokawa with dissipated energy from several experiments and natural observations of fragmented rocks/minerals.


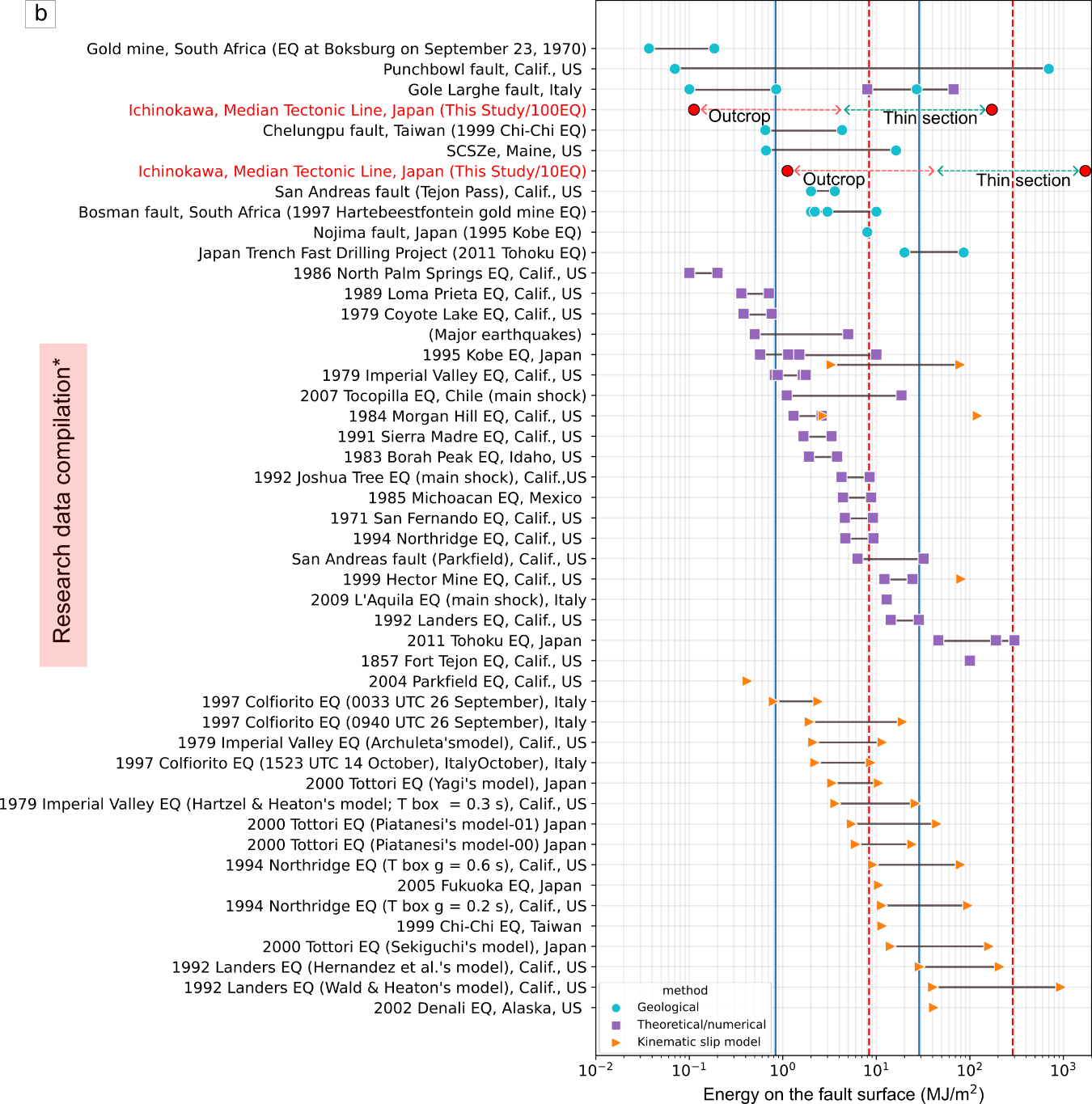


*Supplementary Figures 7.* **(continue)** **Comparison of energy estimates for a single earthquake from several sources compiled by Johnson et al,**^27^. We show the surface energy in Ichinokawa is comparable with other earthquake source volume particularly on outcrop scale. The blue line indicates the average energy for 100EQ scenarios and the red-dashed line for 10EQ scenarios.


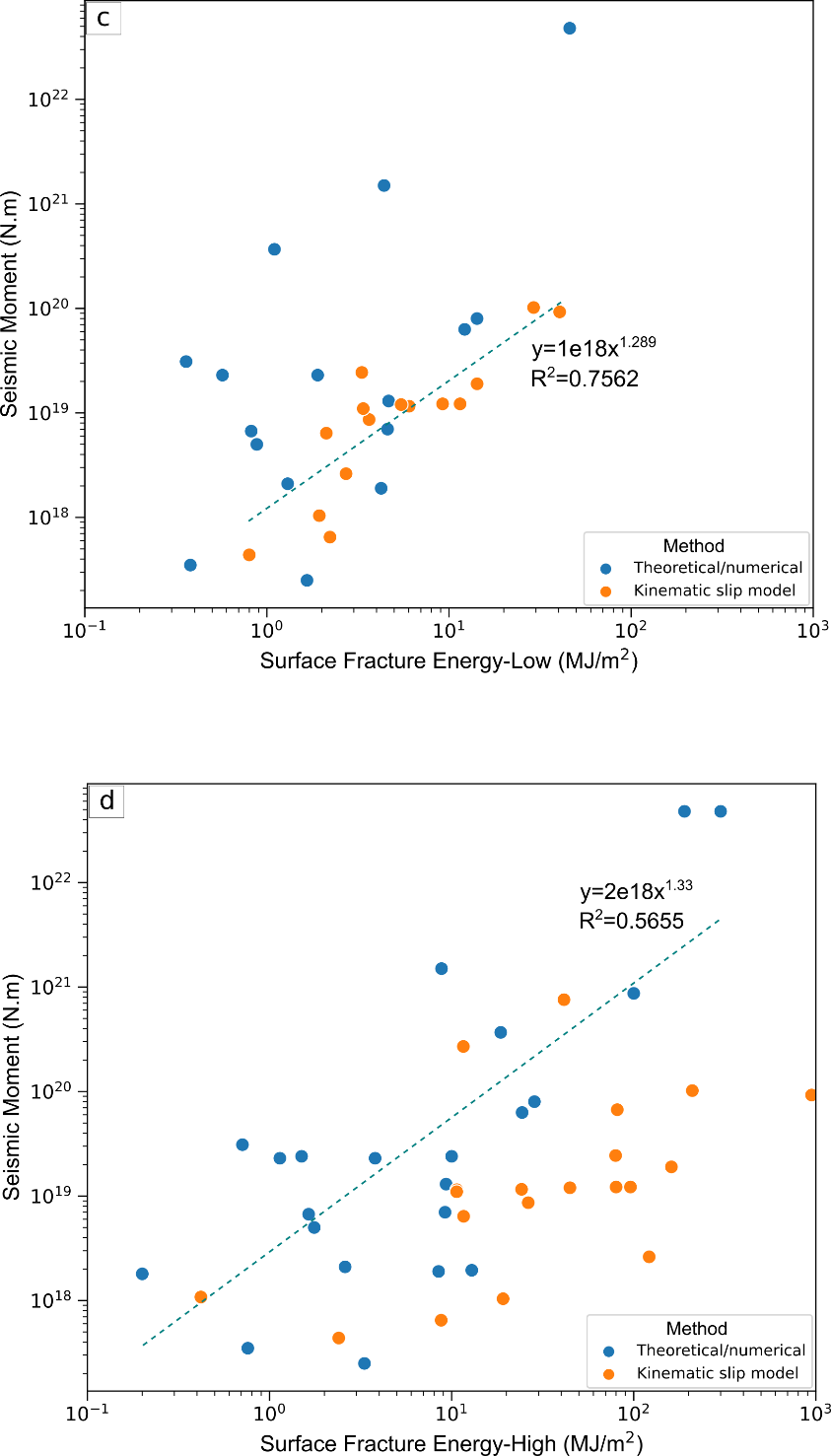


Supplementary Figures 7. **(continue) Surface area energy per unit fault (U_sa_) against Seismic Moment**. From the compiled data, especially the numerical and kinematic model, an attempt to estimate the seismic moment based on its linear relation with surface energy (U_sa_) for (c) low energy and (d) high energy. Estimated seismic moment then translates into moment magnitude using the equation provided by ^28^.


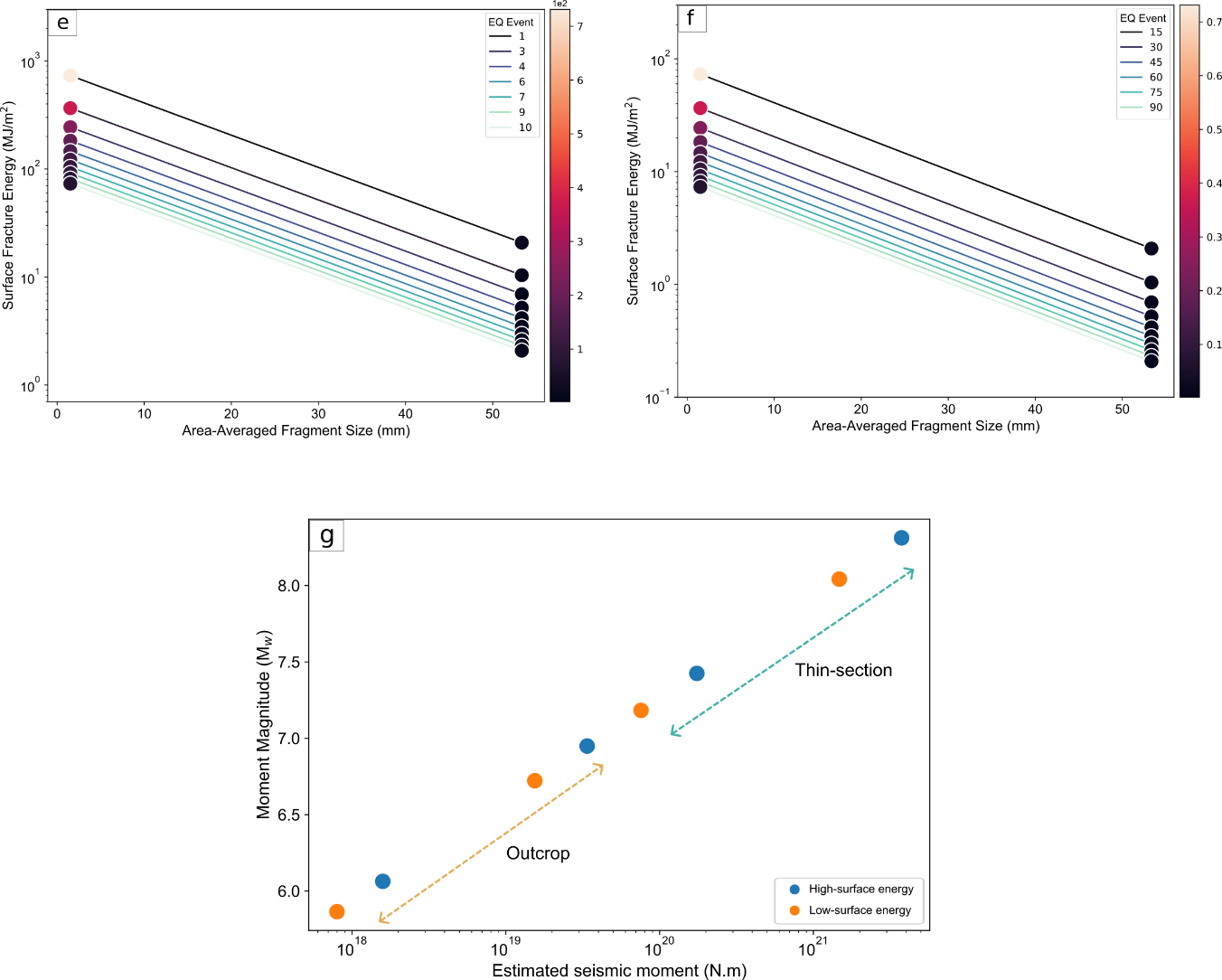


Supplementary Figures 7. **(continue) Surface area energy calculations for scale-integrated PSD data. (e-f)** Estimation of Surface energy per unit fault/damage zone for single earthquake (U_sa_) assuming the total earthquake recurrences of 10 and 100. (g) Calculated earthquake magnitude according to average surface energy.


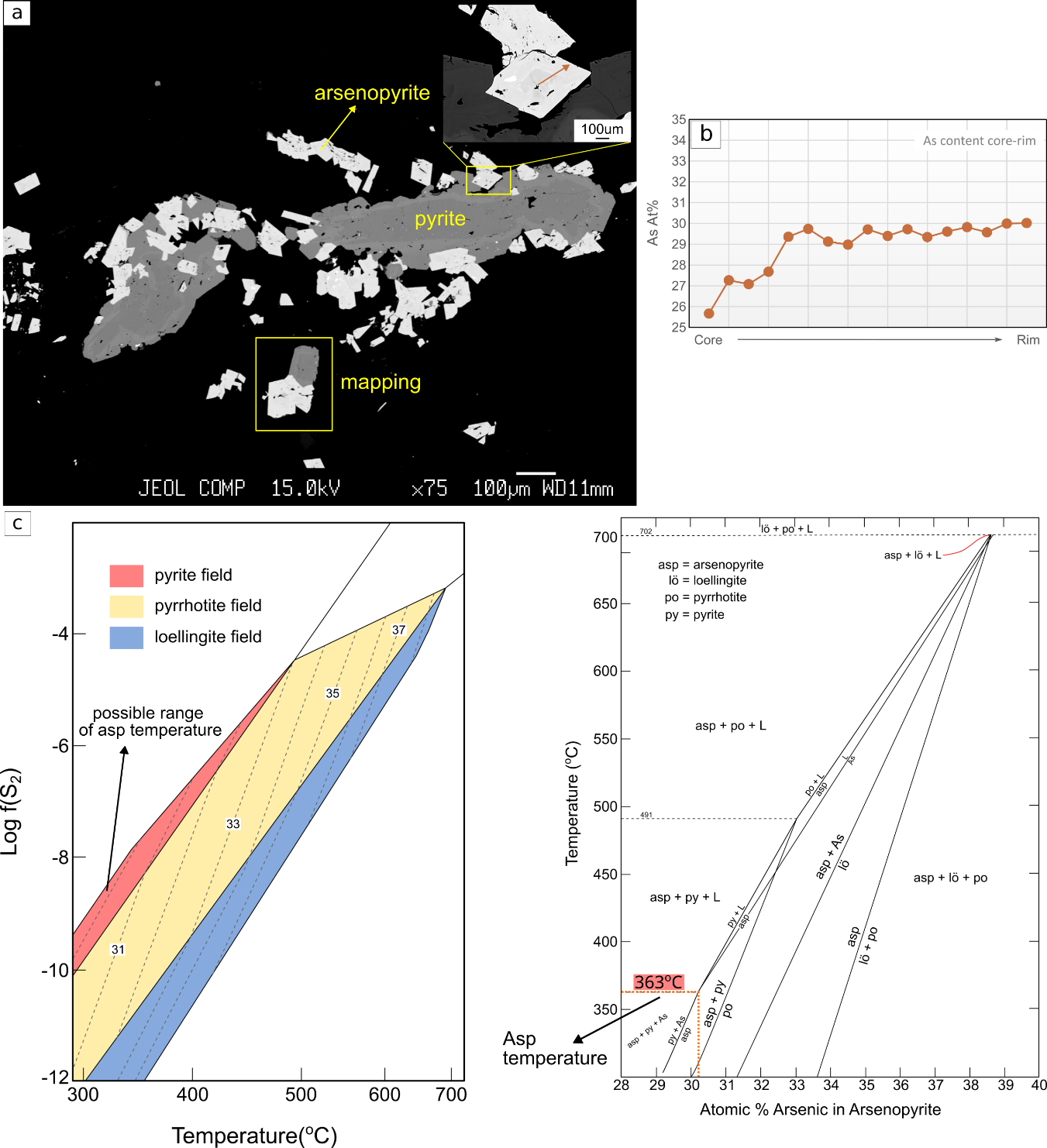


Supplementary Figure 8. **Ore forming fluid temperature from arsenopyrite**. (a) Arsenopyrite texture (b-c) The temperature of formation based on arsenopyrite geothermometry by looking at arsenic concentration and mineral assemblages ^8,9^.


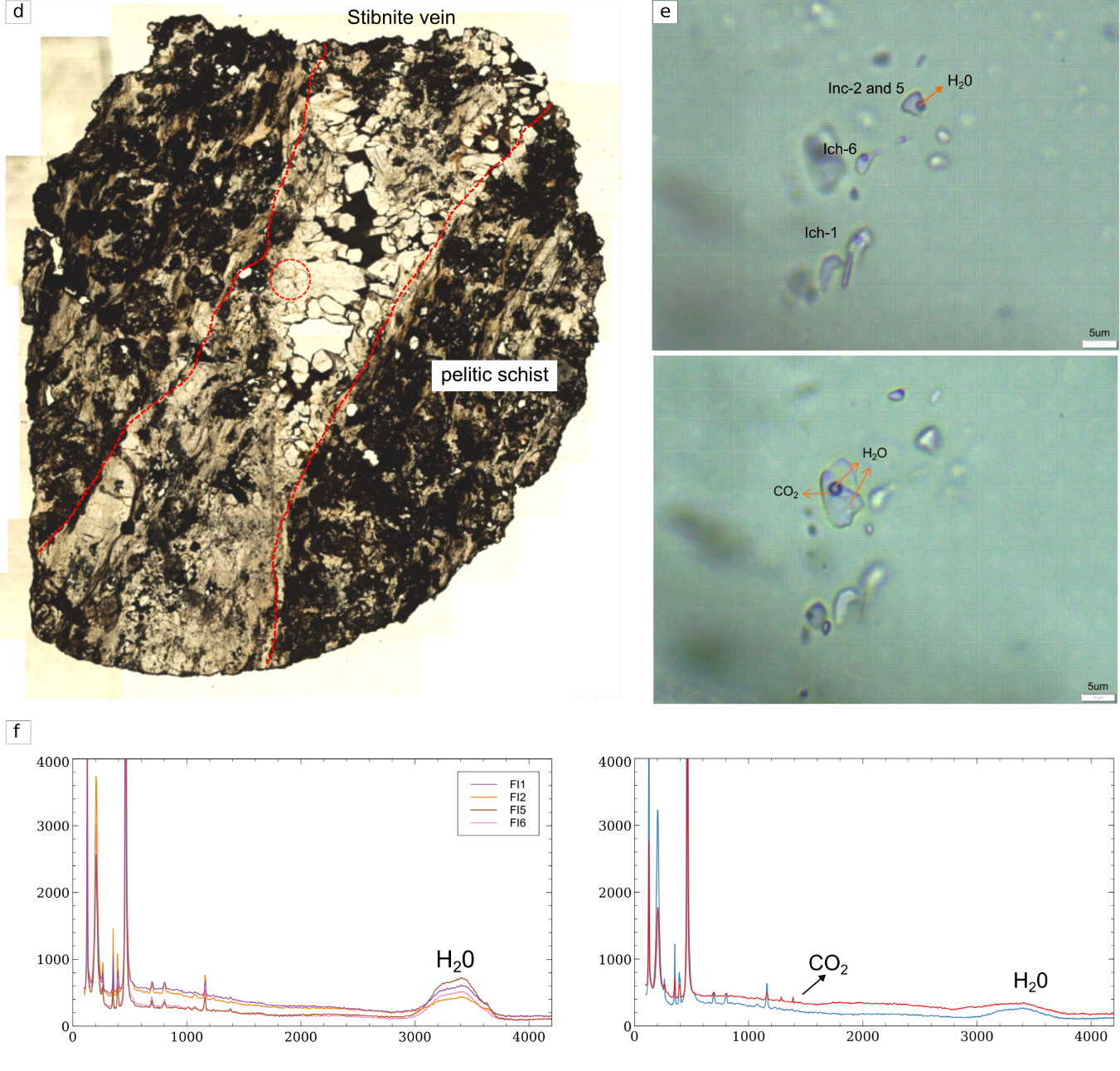


Supplementary Figure 8. **(continue)** **Texture and composition of fluid inclusion.** (d) Red-dashed circle indicates area of interest for fluid inclusion analysis in stibnite vein and characteristic of fluid inclusion in quartz. (e) Petrographics observation of fluid inlclusion show fluid-dominated phases. (f) The raman signal of fluid inclusion shows H_2_O-dominated phases with low concentration of CO_2_.


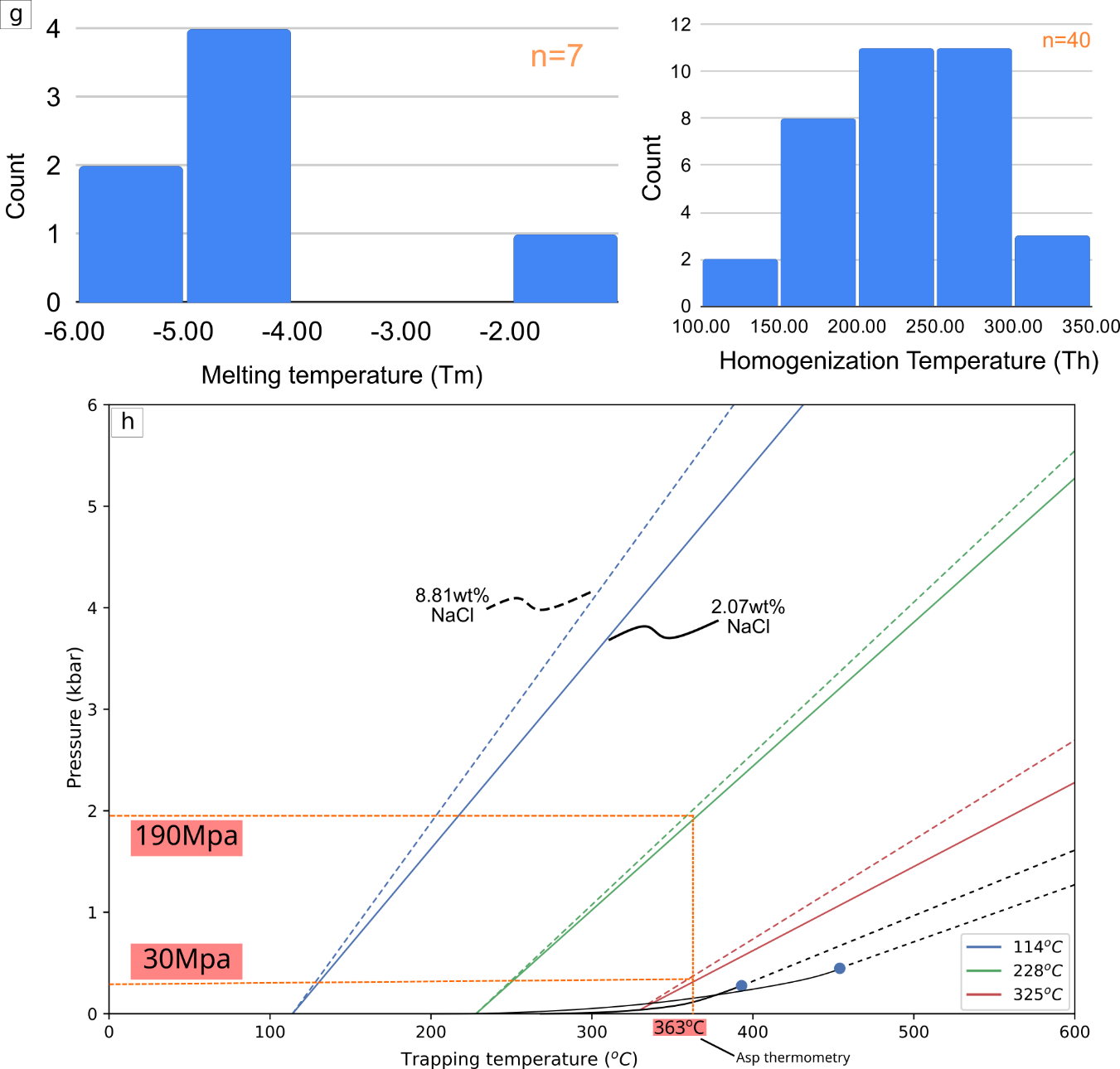


Supplementary Figure 8. **(continue)** **Microthermometry and Isochore modeling of fluid inclusion** (g) Ice melting temperature and homogenization temperature of observed fluid inclusion. (h) Isochore modeling to estimate the pressure of quartz.


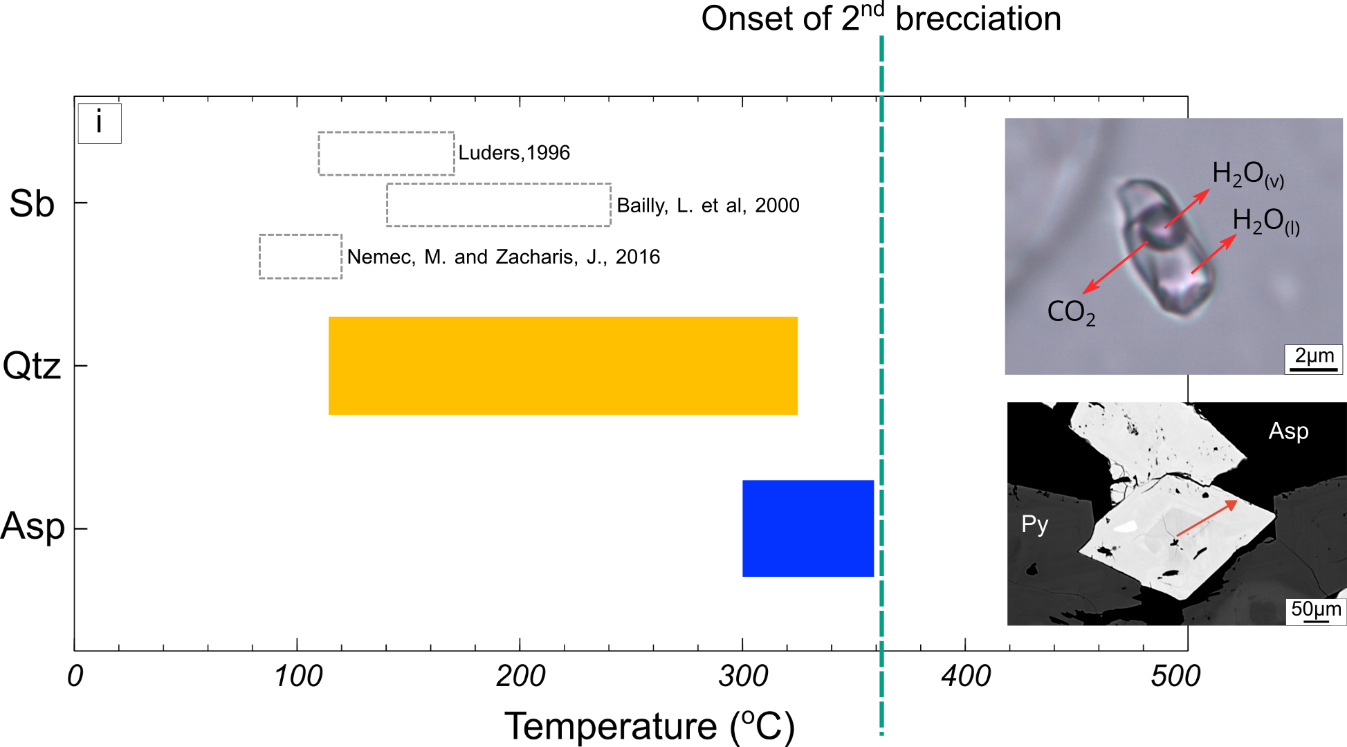


Supplementary Figure 8. **(continue)** **Temperature estimation of ore-forming fluid in Ichinokawa.** (a) Brecciation and mineralization temperature calculated from arsenopyrite and fluid inclusion respectively. The stibnite-forming temperature is derived from several sources. The onset of second brecciation is denoted by the green dashed line.

# Supplementary Tables

Supplementary Tables 1. Summary of fractal dimension (D) within the range of maximum (S_max_) and minimum (S_min_) fragment size. Surface area (L) with calculated Surface area energy density (U_s_) and Surface area energy per fault damage (U_sa_). Including the surface energy for a single earthquake.

| Sample | Breccia width (cm) | D | Smin (mm) | Smax (mm) | L (mm) | Total Us (J/m3) | Total Usa (J/m2) | Usa per EQ (MJ/m2) | |
| --- | --- | --- | --- | --- | --- | --- | --- | --- | --- |
|  |  |  |  |  |  |  |  | 10 EQ | 100 EQ |
| Scale integration | | | | | | | | | |
| Outcrop | - | 1.65 | 20 | 100 | 53.34 | 4.16E+04 | 2.08E+07 | 2.08 | 0.21 |
| Thin section | - | 1.65 | 0.3 | 3.5 | 1.52 | 1.46E+06 | 7.31E+08 | 73.13 | 7.31 |
| Individual PSD | | | | | | | | | |
| Breccia-1 | 30 | 3.56 | 16.13 | 43.35 | 24.30 | 9.12E+04 | 4.56E+07 | 4.56 | 0.46 |
| Breccia-2 | 30 | 1.87 | 13.56 | 45.22 | 26.70 | 8.31E+04 | 4.15E+07 | 4.15 | 0.42 |
| Breccia-2 | 80 | 2.42 | 34.80 | 93.03 | 57.26 | 3.87E+04 | 1.94E+07 | 1.94 | 0.19 |
| Breccia-2 | 80 | 2.35 | 28.16 | 68.65 | 44.41 | 4.99E+04 | 2.50E+07 | 2.50 | 0.25 |
| Breccia-2 | 100 | 2.99 | 64.77 | 160.93 | 98.73 | 2.25E+04 | 1.12E+07 | 1.12 | 0.11 |
| Thin section | - | 1.80 | 0.45 | 0.88 | 0.65 | 3.43E+06 | 1.72E+09 | 172.00 | 17.20 |
| Thin section | - | 1.98 | 0.88 | 2.84 | 1.68 | 1.32E+06 | 6.61E+08 | 66.10 | 6.61 |
| Thin section | - | 2.39 | 1.63 | 3.94 | 2.55 | 8.69E+05 | 4.34E+08 | 43.40 | 4.34 |
| Thin section | - | 1.91 | 0.54 | 1.63 | 1.00 | 2.23E+06 | 1.11E+09 | 111.00 | 11.10 |

Supplementary Tables 1. (Continue). Statistical summary of surface energy per unit fault (U_sa_) for outcrop and thin section scale and estimated magnitude of single earthquake based on number of EQ recuring scenario.

|  | 10 EQ (MJ/m2) | | 100 EQ (MJ/m2) | |
| --- | --- | --- | --- | --- |
|  | Outcrop | Thin section | Outcrop | Thin section |
| Scale integration | | | | |
|  | 2.08 | 73.13 | 0.21 | 7.31 |
| Mw Low | 6.2 | 7.53 | 5.34 | 6.67 |
| Mw High | 6.41 | 7.78 | 5.53 | 6.89 |
| Individual PSD | | | | |
| min | 1.12 | 43.40 | 0.11 | 4.34 |
| max | 45.60 | 172.00 | 4.56 | 17.20 |
| average | 8.36 | 287.00 | 0.84 | 28.70 |
| Mw Low | 6.70 | 8.00 | 5.80 | 7.10 |
| Mw High | 6.90 | 8.30 | 6.00 | 7.40 |

Supplementary Tables 2. **Representative composition of Arsenopyrite from core to rim**. Atomic percentage (At%) is used to determine the temperature in (Supplementary Fig. 2)

|  | Core |  |  |  |  |  |  | Rim |
| --- | --- | --- | --- | --- | --- | --- | --- | --- |
| Fe | 36.46 | 36.05 | 35.99 | 35.71 | 36.02 | 35.71 | 35.26 | 35.32 |
| S | 24.02 | 23.85 | 22.84 | 22.77 | 22.77 | 22.74 | 22.15 | 22.36 |
| As | 39.37 | 39.83 | 43.02 | 41.27 | 42.28 | 41.97 | 42.10 | 42.69 |
| Sb | 0.71 | 0.73 | 0.28 | 0.31 | 0.21 | 0.21 | 0.22 | 0.18 |
| Total (Fe,S,As) | 99.85 | 99.73 | 101.84 | 99.75 | 101.07 | 100.42 | 99.50 | 100.37 |
| Fe (At%) | 33.87 | 33.60 | 33.37 | 33.65 | 33.60 | 33.50 | 33.51 | 33.29 |
| S (At%) | 38.86 | 38.72 | 36.89 | 37.37 | 37.00 | 37.16 | 36.67 | 36.71 |
| As (At%) | 27.27 | 27.68 | 29.74 | 28.98 | 29.40 | 29.34 | 29.82 | 29.99 |

Supplementary Tables 3. **Fluid inclusion (FI) microthermometry.** Th: homogenization temperature, Tm: final ice melting temperature. Most FI show V-L transformation upon heating experiment.

| Quartz Grain | Th (°C) | FI size (µm) | Tm (°C) |
| --- | --- | --- | --- |
| qtz1 | 183 | 3 | - |
| qtz1 | 230 | 1.3 | - |
| qtz1 | 280.8 | 1.5 | - |
| qtz1 | 304.6 | 1.7 | - |
| qtz2 | 172 | 1.695 | - |
| qtz2 |  | 3 | - |
| qtz2 | 190 | 1.745 | - |
| qtz2 | 206.9 | 1 | - |
| qtz2 | 230.7 | 1.07 | - |
| qtz2 | 259 | 1.5 | - |
| qtz2 | 251.9 | 1 | - |
| qtz2 | 276.6 | 1.4 | - |
| qtz2 | 294.5 | 1 | - |
| qtz2 | 304 | 1 | - |
| qtz2 | 325.2 | 1.17 | - |
| qtz3 | 273 | 0.66 | - |
| qtz4 | 295.7 | 0.9 | - |
| qtz5 | 207.7 | 1.84 | - |
| qtz5 | 255 | 2 | - |
| qtz5 | 261.4 | 1.73 | - |
| qtz6 | 238.2 | 1.73 | - |
| qtz7 | 230 | 1 | - |
| qtz7 | 251 | 1.395 | - |
| qtz7 | 243.3 | 1.15 | - |
| qtz8 | 114 | 1 | - |
| qtz8 | 154.1 | 2 | - |
| qtz8 | 182.6 | 3 | - |
| qtz8 | 158 | 5 | -5.2 |
| qtz8 | 221.6 | 1 | -5 |
| qtz8 | 250 | 1 | -5 |
| qtz8 | 227.6 | 2 | -4.6 |
| qtz8 | 120 | 1 | -5 |
| qtz8 | 152 | 3.5 | -1.2 |
| qtz8 | 247.9 | 1.4 | - |
| qtz9 | 190.8 | 2.2 | -5.7 |
| qtz9 | 220 | 1.5 | - |

Supplementary Tables 4. **Dolomite concentration** of the breccia matrix with variation of Fe and Mg as shown in (Supplementary Fig. 1)

|  | dol1 | dol2 | dol3 | dol4 | dol5 | dol6 | dol7 | dol8 | dol9 | dol10 |
| --- | --- | --- | --- | --- | --- | --- | --- | --- | --- | --- |
| CaO | 33.08 | 33.55 | 34.35 | 33.77 | 32.39 | 32.18 | 32.58 | 32.27 | 31.60 | 30.98 |
| MgO | 9.68 | 9.30 | 9.90 | 9.68 | 9.24 | 6.87 | 9.38 | 9.65 | 8.23 | 6.92 |
| FeO | 12.58 | 12.20 | 12.10 | 11.70 | 12.75 | 13.64 | 11.32 | 11.79 | 13.78 | 14.90 |
| Na2O | 0.08 | 0.08 | 0.00 | 0.08 | 0.01 | 0.00 | 0.08 | 0.02 | 0.00 | 0.09 |
| K2O | 0.00 | 0.00 | 0.00 | 0.00 | 0.00 | 0.00 | 0.02 | 0.00 | 0.01 | 0.00 |
| Al2O3 | 0.00 | 0.05 | 0.09 | 0.05 | 0.06 | 0.03 | 0.03 | 0.00 | 0.07 | 0.00 |
| MnO | 0.55 | 0.63 | 0.47 | 0.65 | 1.06 | 2.49 | 1.66 | 1.09 | 0.67 | 0.91 |
| TiO2 | 0.05 | 0.00 | 0.00 | 0.04 | 0.00 | 0.02 | 0.00 | 0.00 | 0.00 | 0.00 |
| Total | 56.03 | 55.80 | 56.91 | 55.97 | 55.51 | 55.24 | 55.08 | 54.82 | 54.36 | 53.80 |
| Ca | 3.48 | 3.55 | 3.55 | 3.55 | 3.46 | 3.55 | 3.50 | 3.47 | 3.48 | 3.50 |
| Mg | 1.42 | 1.37 | 1.42 | 1.41 | 1.37 | 1.05 | 1.40 | 1.44 | 1.26 | 1.09 |
| Fe | 1.03 | 1.01 | 0.98 | 0.96 | 1.06 | 1.17 | 0.95 | 0.99 | 1.19 | 1.32 |
| Na | 0.02 | 0.02 | 0.00 | 0.02 | 0.00 | 0.00 | 0.01 | 0.00 | 0.00 | 0.02 |
| K | 0.00 | 0.00 | 0.00 | 0.00 | 0.00 | 0.00 | 0.00 | 0.00 | 0.00 | 0.00 |
| Al | 0.00 | 0.01 | 0.01 | 0.01 | 0.01 | 0.00 | 0.00 | 0.00 | 0.01 | 0.00 |
| Mn | 0.05 | 0.05 | 0.04 | 0.05 | 0.09 | 0.22 | 0.14 | 0.09 | 0.06 | 0.08 |

Supplementary Tables 4. **(continue)**

|  | dol11 | dol12 | dol13 | dol14 | dol15 | dol16 | dol17 | dol18 | dol19 | dol20 |
| --- | --- | --- | --- | --- | --- | --- | --- | --- | --- | --- |
| CaO | 30.64 | 31.13 | 31.14 | 31.08 | 31.40 | 31.47 | 31.31 | 30.90 | 31.71 | 32.73 |
| MgO | 6.51 | 6.56 | 6.70 | 7.57 | 8.06 | 8.33 | 8.38 | 9.15 | 9.22 | 8.81 |
| FeO | 16.05 | 16.31 | 15.61 | 13.39 | 13.12 | 12.56 | 11.97 | 12.75 | 11.66 | 13.32 |
| Na2O | 0.00 | 0.00 | 0.00 | 0.00 | 0.00 | 0.30 | 0.03 | 0.00 | 0.00 | 0.00 |
| K2O | 0.00 | 0.00 | 0.00 | 0.01 | 0.00 | 0.00 | 0.00 | 0.01 | 0.00 | 0.02 |
| Al2O3 | 0.00 | 0.00 | 0.00 | 0.00 | 0.00 | 0.01 | 0.00 | 0.04 | 0.00 | 0.00 |
| MnO | 1.07 | 1.44 | 1.46 | 1.57 | 1.36 | 1.68 | 1.62 | 1.73 | 1.46 | 1.55 |
| TiO2 | 0.02 | 0.01 | 0.00 | 0.00 | 0.00 | 0.00 | 0.01 | 0.00 | 0.02 | 0.02 |
| Total | 54.28 | 55.44 | 54.90 | 53.62 | 53.95 | 54.35 | 53.31 | 54.57 | 54.08 | 56.45 |
| Ca | 3.46 | 3.45 | 3.47 | 3.50 | 3.49 | 3.47 | 3.50 | 3.37 | 3.47 | 3.47 |
| Mg | 1.02 | 1.01 | 1.04 | 1.19 | 1.25 | 1.28 | 1.31 | 1.39 | 1.40 | 1.30 |
| Fe | 1.42 | 1.41 | 1.36 | 1.18 | 1.14 | 1.08 | 1.05 | 1.09 | 1.00 | 1.10 |
| Na | 0.00 | 0.00 | 0.00 | 0.00 | 0.00 | 0.06 | 0.01 | 0.00 | 0.00 | 0.00 |
| K | 0.00 | 0.00 | 0.00 | 0.00 | 0.00 | 0.00 | 0.00 | 0.00 | 0.00 | 0.00 |
| Al | 0.00 | 0.00 | 0.00 | 0.00 | 0.00 | 0.00 | 0.00 | 0.01 | 0.00 | 0.00 |
| Mn | 0.10 | 0.13 | 0.13 | 0.14 | 0.12 | 0.15 | 0.14 | 0.15 | 0.13 | 0.13 |

# References

1. Okamoto, A. & Sekine, K. Textures of syntaxial quartz veins synthesized by hydrothermal experiments. *J. Struct. Geol.* **33**, 1764–1775 (2011).

2. Watt, G. R., Wright, P., Galloway, S. & McLean, C. Cathodoluminescence and trace element zoning in quartz phenocrysts and xenocrysts. *Geochim. Cosmochim. Acta* **61**, 4337–4348 (1997).

3. Okamoto, A., Saishu, H., Hirano, N. & Tsuchiya, N. Mineralogical and textural variation of silica minerals in hydrothermal flow-through experiments: Implications for quartz vein formation. *Geochim. Cosmochim. Acta* **74**, 3692–3706 (2010).

4. Rusk, B. & Reed, M. Scanning electron microscope-cathodoluminescence analysis of quartz reveals complex growth histories in veins from the Butte porphyry copper deposit, Montana. *Geology* **30**, 727–730 (2002).

5. Saishu, H., Okamoto, A. & Tsuchiya, N. The significance of silica precipitation on the formation of the permeable-impermeable boundary within Earth’s crust. *Terra Nova* **26**, 253–259 (2014).

6. Akçay, M., Özkan, H. M., Moon, C. J. & Spiro, B. Geology, mineralogy and geochemistry of the gold-bearing stibnite and cinnabar deposits in the Emirli and Hali{dotless}köy areas (Ödemiş, İzmir, West Turkey). *Ore Geol. Rev.* **29**, 19–51 (2006).

7. Bellot, J. P., Lerouge, C., Bailly, L. & Bouchot, V. The biards Sb-Au-bearing shear zone (Massif Central, France): An indicator of crustal-scale transcurrent tectonic guiding Late Variscan Collapse. *Econ. Geol.* **98**, 1427–1447 (2003).

8. Kretschmar, U. & Scott, S. D. Phase relations involving arsenopyrite in the system Fe-As-S and their application. *Iraduit Par J.* **14**, 364–386 (1976).

9. Sharp, Z. D., Essene, E. J. & Kelly, W. C. A reexamination of the arsenopyrite geothermometer/ geobarometer; pressure considerations and corrections. *AGU 1984 Spring Meet.* **23**, 290 (1984).

10. Agroli, G., Okamoto, A., Uno, M. & Tsuchiya, N. Transport and evolution of supercritical fluids during the formation of the erdenet CU–MO deposit, Mongolia. *Geosci. Switz.* **10**, (2020).

11. Wark, D. A. & Watson, E. B. TitaniQ: A titanium-in-quartz geothermometer. *Contrib. Mineral. Petrol.* **152**, 743–754 (2006).

12. Dill, H. G. The ‘chessboard’ classification scheme of mineral deposits: Mineralogy and geology from aluminum to zirconium. *Earth-Sci. Rev.* **100**, 1–420 (2010).

13. Sakakibara, M. & Isono, Y. Middle Miocene thermal metamorphism due to the infiltration of high-temperature fluid in the Sanbagawa metamorphic belt, southwest Japan. *Contrib. Mineral. Petrol.* **125**, 341–358 (1996).

14. Cheval-Garabédian, F., Faure, M., Marcoux, E., Gouin, J. & Picault, M. The La Bellière gold and antimony district (French Armorican Massif): A two-stage evolution model controlled by Variscan strike-slip tectonic. *Ore Geol. Rev.* **125**, 103681 (2020).

15. Sakakibara, M., Umeki, M. & Cartwright, I. Isotopic evidence for channeled fluid flow in low-grade metamorphosed Jurassic accretionary complex in the Northern Chichibu belt, western Shikoku, Japan. *J. Metamorph. Geol.* **25**, 383–400 (2007).

16. Bailly, null, Bailly, L., Bouchot, V., Bény, C. & Milési, J. P. Fluid inclusion study of stibnite using infrared microscopy: An example from the Brouzils antimony deposit (Vendee, Armorican massif, France). *Econ Geol* **95**, 221–226 (2000).

17. Němec, M. & Zachariáš, J. The Krásná Hora, Milešov, and Povy Sb-Au ore deposits, Bohemian Massif: mineralogy, fluid inclusions, and stable isotope constraints on the deposit formation. *Miner. Deposita* **53**, 225–244 (2018).

18. Krupp, R. E. Solubility of stibnite in hydrogen sulfide solutions, speciation, and equilibrium constants, from 25 to 350°C. *Geochim. Cosmochim. Acta* **52**, 3005–3015 (1988).

19. Li, W. *et al.* Textures and trace element signatures of pyrite and arsenopyrite from the gutaishan Au–Sb deposit, south China. *Miner. Deposita* **54**, 591–610 (2019).

20. Shimizu, T. Fluid Inclusion Studies of Comb Quartz and Stibnite at the Hishikari Au–Ag Epithermal Deposit, Japan. *Resour. Geol.* **68**, 326–335 (2018).

21. Wagner, T. & Cook, N. J. Late-Variscan antimony mineralisation in the Rheinisches Schiefergebirge, NW Germany: Evidence for stibnite precipitation by drastic cooling of high-temperature fluid systems. *Miner. Deposita* **35**, 206–222 (2000).

22. Bakker, R. J. AqSo_NaCl: Computer program to calculate p-T-V-x properties in the H2O-NaCl fluid system applied to fluid inclusion research and pore fluid calculation. *Comput. Geosci.* **115**, 122–133 (2018).

23. A’xiang, H. & Jiantang, P. Fluid inclusions and ore precipitation mechanism in the giant Xikuangshan mesothermal antimony deposit, South China: Conventional and infrared microthermometric constraints. *Ore Geol. Rev.* **95**, 49–64 (2018).

24. Wilkinson, J. J. Fluid inclusions in hydrothermal ore deposits. *Lithos* **55**, 229–272 (2001).

25. Amagai, T. *et al.* Silica nanoparticles produced by explosive flash vaporization during earthquakes. *Sci. Rep.* **9**, 1–9 (2019).

26. Weatherley, D. K. & Henley, R. W. Flash vaporization during earthquakes evidenced by gold depositsWeatherley, D. K., & Henley, R. W. (2013). Flash vaporization during earthquakes evidenced by gold deposits. Nature Geoscience, 6(4), 294–298. https://doi.org/10.1038/ngeo1759. *Nat. Geosci.* **6**, 294–298 (2013).

27. Johnson, S. E., Song, W. J., Vel, S. S., Song, B. R. & Gerbi, C. C. Energy Partitioning, Dynamic Fragmentation, and Off-Fault Damage in the Earthquake Source Volume. *J. Geophys. Res. Solid Earth* **126**, e2021JB022616 (2021).

28. Kanamori, H. & Brodsky, E. E. The physics of earthquakes. *Rep. Prog. Phys.* **67**, 1429 (2004).
